## [Decision Letter · Decision Letter 0]

24 Nov 2021

Dear Dr. Alper,

Thank you very much for submitting your manuscript "Light chain 2 is a Tctex-type related axonemal dynein light chain that regulates directional ciliary motility in Trypanosoma brucei" for consideration at PLOS Pathogens. As with all papers reviewed by the journal, your manuscript was reviewed by members of the editorial board and by several independent reviewers. In light of the reviews (below this email), we would like to invite the resubmission of a significantly-revised version that takes into account the reviewers' comments.

We cannot make any decision about publication until we have seen the revised manuscript and your response to the reviewers' comments. Your revised manuscript is also likely to be sent to reviewers for further evaluation.

Sincerely,

David Sacks

Section Editor

PLOS Pathogens

David Sacks

Section Editor

PLOS Pathogens

Kasturi Haldar

Editor-in-Chief

PLOS Pathogens

orcid.org/0000-0001-5065-158X

Michael Malim

Editor-in-Chief

PLOS Pathogens

orcid.org/0000-0002-7699-2064

Reviewer's Responses to Questions

**Part I - Summary**

Reviewer #1: This paper describes the structure and function of a light-chain dynein-related protein in Trypanosoma brucei that the authors refer to as TbLC2. The authors investigate its localization and model its structure with a variety of standard methods. The effect of TbLC2 on motility is described at some length, in a series of experiments in which the authors use FLAM3 knockdown cells to enable better visualization of flagellar waveforms. The role of TbLC2 in regulating flagellar beating is discussed and compared to the role played by structurally similar proteins in other model microorganisms such as C. reinhardtii.

The authors begin by showing computational predictions of the structure of TbLC2, using some standard modelling frameworks – this is not my area of expertise, but the results seemed solid. Next the localization of the protein of interest was examined using fluorescence microscopy, and the authors have obtained some particularly convincing images of the adjacent localization of the paraflagellar rod (PFR) and LC2. The role of motility in assisting with the replicative cycle has been examined elsewhere, and the authors investigate this using shaken/unshaken cultures. They find that impairment of motility by LC2 is associated with larger cell clusters when the cultures are not shaken. Electron microscopy data shows that axoneme assembly is not contingent on TbLC2, unlike the analogous case in C. reinhardtii. Cells sediment more quickly in the case of the TbLC2 knockdown, even though the flagellar beat frequency is higher (measured by optical tweezers). The authors conduct a detailed video cell-tracking motility assay to determine the curvilinear speed and total displacement of the cells after ten seconds. Lastly, they perform a single-cell image analysis assay to investigate the asymmetry of the flagellar beat and fraction of ‘dwell time’ in either tip-to-base or base-to-tip propagation in the case of FLAM3 and LC2 knockdown.

This is a long and careful study, taking in many techniques and sets of experiments. Overall, I think that the paper is solid, and that the conclusions are justified by the results. The findings are novel (to my knowledge) and the execution is highly competent. I have a few fairly minor issues with the presentation of data, but my main complaint is that the manuscript is overlong, and repetitive in places. Some overlap between results/discussion/conclusion is to be expected (and I appreciate that this something of a matter of taste), but it’s tiring for the reader to encounter the same statements (e.g. those about flagellar asymmetry) in several places, in a similar context throughout a 56-page manuscript. I should also note that this only affects some parts - the optical tweezers section is succinct, for example - but the sections on the flagellar waveform in particular stuck out.

Reviewer #2: In this work, Godar and colleagues characterize a dynein light chain (LC2) in T. brucei using a variety of techniques, including loss-of-function experiments and live-cell imaging of laser-tweezer trapped cells to better understand the mechanics of flagellar beat in the absence of the protein. The manuscript as currently configured reads more like two separate papers (LC2 phenotypic characterization, flagellar beat studies) and because of this is quite long. There are a few places where the authors have broken from nomenclature conventions that make following the work confusing. For example, in my understanding the "delta" nomenclature the authors use to label their RNAi cell lines should only be used for true knockouts, where the gene itself has been removed from the cells, not just suppressed by RNAi. Also, there are multiple types of overexpression being used to modulate LC2- some tet-inducible, others not, that are not clearly detailed in the text.

**Part II – Major Issues: Key Experiments Required for Acceptance**

Reviewer #1: (No Response)

Reviewer #2: One of my biggest criticisms is that the authors do not really explain why they are doing essentially all their experiments in a FLAM3 RNAi background until the discussion. Much of the LC2 characterization is done without really regarding what the additional effect of FLAM3 depletion could be having on the cells when LC2 is perturbed. This needs to be clearly stated and accounted for. Is it not possible to do the biophysical analysis of the flagellar beat with an attached flagella?

The approach that the authors employ to "rescue" their LC2 RNAi produces a significant growth defect on its own, which makes it difficult to see it as a true rescue experiment. Even though the authors clearly show that it has a deleterious effect, they employ it as a control in their flagellar beat experiments. If overexpression is toxic, the authors could switch to an RNAi strategy where a portion of the coding sequence is recoded and then target the native sequence for RNAi. That would allow them to create an RNAi-insensitive allele at the native locus, which should have more wild-type expression levels. The degree of overexpression between the pXS2 O/E and the pLEW O/E should also be characterized by western blotting.

The phenotypic characterization of LC2 breaks with some of the best-practice approaches used in the field, such as the categorization of all non-1N1K cells as abnormal, instead of looking at the range of DNA states seen in a healthy, dividing cell population. Also, the simplest analysis where just LC2 is depleted (rather than both FLAM3/LC2) should have been the starting point of the analysis, especially since the "rescue" cells where LC2 is likely to be significantly overexpressed are likely to have their own phenotypic issues.

Considering that the flagellum is usually mostly attached in procyclic cells, it seems that this arrangement, which includes the FAZ and the transmission of the force from the flagellar beat into the MTs that surround the cell body, would have some effect on some of the parameters that the authors are measuring in their imaging. In some ways, this feels like a fairly artificial system for studying the function of LC2 because the cells have been perturbed already from their natural state by FLAM3 depletion. In other words, how do we know that LC2 function in an essentially detached flagellum reflects its function in an attached flagellum, which is its natural state in this form of the parasite?

**Part III – Minor Issues: Editorial and Data Presentation Modifications**

Reviewer #1: Line 61: ‘underlie’

Line 144: ‘characterized by a higher’ (missing ‘a’)

The introduction of FLAM3 knockdown is very brief and the unfamiliar reader might not appreciate the significant morphological change to which it gives rise. It would be good to expand the text a little around line 209 given the central utility of this modification.

Could the authors be a bit clearer about the nomenclature in line 245? How about something like: “‘NN’ and ‘MK’ are used to represent cells with N nuclei and M kinetoplasts”? Otherwise the reference to N>2 is a bit confusing considering that you talk about 2K1N later in the figure caption.

In the optical tweezers assay, Fig. 6 is a bit confusing. The cartoon in Fig. 6A and Fig. 6B show a cell with its anterior-posterior line more-or-less in the focal plane. Movie S1A shows the cell oriented at 90 degrees to this and aligned with the laser beam axis (you can see the anterior tip far from the focal plane, I’d guess towards the observer). The latter is what I would expect for a cell trapped for any length of time; it has a higher refractive index than its environment, so the cell body ‘wants’ to sit inside the laser beam as much as possible. This would tally with the 1-2Hz frequency observed, and it seems. Is that right, and if so, could you correct the figure?

The figure numbering isn’t consistently referenced. In line 484 (and subsequently), the authors refer to Fig.7 when I think they mean Fig. 5. Could they check that the figures are referenced correctly?

The use of ‘velocity’ and ‘speed’ are misleading, as these are (mathematically speaking) vector and scalar versions of the same quantity. The authors could leave ‘speed’ as is, but replace ‘velocity’ with ‘total displacement after 10 seconds’, modifying the relevant quantities

Please soften the statement in line 508-510; the ciliary waveform is presumably different in LC2 knockdown, and could be argued to give a smaller net force (as is observed through lower net speed).

Line 609: I’d suggest 'bias' (rather than ‘biasedness’)

The data in lines 616-619 would be better presented in a table, rather than in-line.

Lines 731-733: Chlamydomonas cells rely on balancing the torque from one flagellum with that from the second in order to move in a straight line; removing one flagellum doesn’t change the direction of the beating wave, it makes cells rotate more-or-less in place because the torque isn’t balanced any more. I’d suggest revising this sentence. Also, it’s interesting to note that Chalmydomonas *do* occasionally have a quasi-symmetric beating mode – the so-called ‘shock response’ (e.g. Rüffer and Nultsch, Botanica Acta 108 p.255, 1995).

Line 770: I liked the experiments where the samples were agitated, and this adds weight to the hypothesis about cell replication, but the *local* fluid shear rates in an agitated culture are probably much less than those experienced by a cell as it tries to break free from a neighbor so I’m not surprised that the agitation wasn’t sufficient to release cells.

Lines 842-845: I agree that detaching the cilium from the cell body makes it easier to see what’s going on, but the ciliary waveform is the product of dynein activity and passive loads, including the PFR and the viscous load of the cell body. In this light, it seems unlikely that a FLAM3 knockdown simply ‘amplifies’ the flagellar waveforms – the flagellum is under different mechanical constraints, and it’s not clear how general these results will be.

Reviewer #2: Line 196-197: To characterize the localization of LC2, one could use antibodies against axonemal components, such as pan-centrin antibody 20H5, for a direct comparison.

Line 213: Considering that pXS2 employs the PARP promoter, which is the strongest native promoter known in proyclics, it is very likely that LC2-eGFP levels dwarf the endogenous pool of untagged protein- this could be checked by qPCR at least. It is hard to explain why the eGFP-tagged protein doesn't incorporate well in to the axoneme in the presence of the WT protein. Do the authors show that this is actually the case?

Line 209-219: Could more directly check for coloc of LC2 with axoneme with detergent extraction of cells, to remove cytosolic pool, then stain cells for an axonemal marker protein.

Line 233: What is the rationale for studying the LC2 RNAi phenotype in the FLAM3 RNAi background? It makes sense in terms of their localization strategy, but the simplest thing would be to look at the effect of just LC2 RNAi before including the depletion of a second protein.

Line 273: The method used to describe "normal" DNA states does not account for dividing cells (1N2K, 2N2K), which frequently make up >20% of the population. Changes in the distribution of cell cycle stages should be reported and could reflect important aspects of the RNAi phenotype. Grouping everything that is not 1N1K into one category will miss a lot of important data.

Line 293: The production of detached flagella in the LC2 phenotype suggests a defect in the formation of a new FAZ, which would cause issues with placement of the cleavage furrow. This would be an interesting result that arises from depleting a protein that localizes primarily to the flagellum. FAZ state could be checked with a FAZ antibody such as L3B2.

Line 302: Why is this analysis being done in the FLAM3/LC2 dual RNAi, if you are just trying to look at the function of LC2?

Line 384-385: It should be noted that a cell with 3 kinetoplasts and one nucleus would be aberrant under any circumstance, not just in terms of their arrangement within a single cell.

Line 350: The similarities in the growth rates at early stages could be attributable to delays in the turnover of the LC protein. This could be tested by western blotting of the RNAi and control samples to look for when the LC2 levels decline. If tagging is an issue qPCR could be used as an alternative to look for loss of mRNA.

Line 354: I find it curious that there is no change in growth rate between the shaken and unshaken FLAM3/LC2 RNAi cells, considering that the shaking limited the amount of cell clustering. How were the cell counts being done?

Line 379-380: In terms of LC2, what is the difference in expression levels between the "LC2 OE" and "Rescued" cells? Are they both pXS2 overexpressors? The nomenclature here is not clear.

Line 378: Is this overexpression constitutive or tet-inducible? It's not clear but since they are talking about induction in later paragraphs I assume it's tet-inducible? From checking the M&M I realize that there are pXS2 and pLEW100 versions of the overexpression being used- it should be made very clear to distinguish between the two. If a pXS2 O/E is being compared to a pLEW100 in this case, an anti-GFP western could be used to see if one is expressing more than the other. In my experience, pLEW100 O/E tend to be higher than a constitutive O/E like pXS2 because with tet induction long-term cell viability is not necessary, so you can achieve levels of protein expression that you can't with constitutive expression.

Line 1003-1010: From digging in the materials and methods I now see that the depletion of LC2 was checked using qPCR. This should be mentioned in the results section. Parts of the M&M read more like a Results section- I don't think there should be data here that is not mentioned in the rest of the work?

Line 448: "Overexpressing TbLC2::eGFP in the FLAM3/rescued cells..." Is this pXS2 overexpression that is implied by "rescued" or some additional overexpression? This is very confusing.

Line 504-507: If the overexpression of the LC2::eGFP is causing motility defects, I don't understand how it can be called a "rescue" control. Technically, an equally reasonable interpretation is that the RNAi has an off-target effect that cannot be compensated for by the eGFP allele.

PLOS authors have the option to publish the peer review history of their article (what does this mean?). If published, this will include your full peer review and any attached files.

Reviewer #1: No

Reviewer #2: No
---

## [Decision Letter · Decision Letter 1]

27 Apr 2022

Dear Dr. Alper,

Thank you very much for submitting your manuscript "Light chain 2 is a Tctex-type related axonemal dynein light chain that regulates directional ciliary motility in Trypanosoma brucei" for consideration at PLOS Pathogens. As with all papers reviewed by the journal, your manuscript was reviewed by members of the editorial board and by several independent reviewers. The reviewers appreciated the attention to an important topic. Based on the reviews, we are likely to accept this manuscript for publication, providing that you modify the manuscript according to the review recommendations.

Both reviewers found the revised manuscript to be considerably improved. As you will see, however, concerns remain. I am in agreement with both reviewers that the manuscript remains too long and focuses on some analyses that are not the most relevant, namely cell division and morphological defects observed in the FLAM3-LC2 double knockdown. Such cell division, morphology and organelle distribution defects have been established previously for several flagellum and motility mutants, so observing these defects upon simultaneous knockdown of two flagellar proteins is not novel. Moreover, as pointed out by reviewer 2, the rationale for using the double mutant in this case is not adequate, and it is unclear whether exacerbation of defects reflects a relevant interaction between LC2 and FLAM3, or complex pleotropic effects in the double mutant that cannot be reliably traced directly to LC2 function. This all detracts from the very important and novel work presented with detailed analysis of flagellar motility, for which the FLAM3 knockdown background is justified.

Reviewer 2 outlines a clear path forward with specific recommendations for removing less relevant information. I am recommending that you follow reviewer 2 recommendations. If desired, one could include a summary of the results as supplemental and succinctly indicate in the text something to indicate that the FLAM3-LC2 double knockdown exhibits morphology and cell division defects observed in other flagellum mutants and it is unclear why such defects are exacerbated in the double knockdown while mostly not observed in the LC2 single knockdown.

Sincerely,

Kent L. Hill

Associate Editor

PLOS Pathogens

David Sacks

Section Editor

PLOS Pathogens

Kasturi Haldar

Editor-in-Chief

PLOS Pathogens

orcid.org/0000-0001-5065-158X

Michael Malim

Editor-in-Chief

PLOS Pathogens

orcid.org/0000-0002-7699-2064

Reviewer Comments (if any, and for reference):

Reviewer's Responses to Questions

**Part I - Summary**

Reviewer #1: This paper describes the structure and function of a light-chain dynein-related protein in Trypanosoma brucei that the authors refer to as TbLC2. The authors investigate its localisation and model its structure with a variety of standard methods. The effect of TbLC2 on motility is described at some length, in a series of experiments in which the authors use FLAM3 knockdown cells to enable better visualisation of flagellar waveforms. The role of TbLC2 in regulating flagellar beating is discussed and compared to the role played by structurally similar proteins in other model microorganisms such as C. reinhardtii. (comments on novelty and significance are unchanged from my previous review - the work is novel, significant and well-executed)

Reviewer #2: See below

**Part II – Major Issues: Key Experiments Required for Acceptance**

Reviewer #1: None

Reviewer #2: In this revision, the authors have addressed some of the points brought up during the original review, but I have substantial concerns about the work as it is currently formulated. First and foremost, the clarification about the use of the FLAM3 co-RNAi to study the LC2 RNAi phenotype makes sense for the laser tweezer and perhaps some of the motility assays- it is necessary to unmask the flagellum from the cell body. However, no justification is provided for the study of the LC2 FLAM3 co-RNAi for the cell division and morphology data provided in the paper. FLAM3 is a component of the flagellum side of the FAZ; LC2 is an outer arm dynein- these two structures are not likely to be in contact with one another or have a direct effect on each other- the authors do not provide any evidence for this. The idea appears to revolve around synthetic effects that arise when both proteins are depleted. The main reason for including the FLAM3 RNAi appears to be that it exacerbates the very mild effects of LC2 RNAi- considering that there is no obvious or proposed connection between these proteins, it is difficult to understand how this is justified- it is certainly not explored in this paper. It is possible that performing RNAi on two other proteins with mild phenotypes would produce a stronger effect, which may arise due to complex pleiotropic effects. The authors attribute the stronger effects of the dual depletion as strictly unmasking hidden function of LC2, showing its true importance, when the same case can be made for FLAM3. If they are interested in the synthetic effect, they need to consider the contributions of both proteins.

I strongly suggest that the authors remove all of the cell division and morphology data from the paper that pertains to the dual FLAM3/LC2 RNAi. This would significantly streamline the paper- I will try to propose what could be shortened or omitted below. The strongest part of the paper, and where the dual-RNA can be justified, is the analysis of the paper starting on line 449. The construction of the cell lines, localization of LC2, and the RNAi phenotypes specific to LC2 can all be included as a lead-in to this work.

**Part III – Minor Issues: Editorial and Data Presentation Modifications**

Reviewer #1: The authors have significantly revised several aspects of their manuscript in this resubmission, and I thank them for clearly indicating the aspects that have changed. This is helpful in the context of a manuscript that is still rather lengthy: ~200 pages now, although I note that this includes the rebuttal. The clarification of the cell geometry in the optical tweezers experiment is great, and the additional context around Chlamydomonas motility is welcome. I still take issue with a couple of aspects, but these are now largely a matter of taste due to the authors' careful clarifications, and shouldn't prevent publication:

- The order-of magnitude shear stress calculation is interesting, but I'm not sure it's the right calculation. Warboys et al. (the reference cited) study the flow of fluid over a surface attached epthelial layer, but I believe that the T. brucei are in suspension? If so, that reduces the shear rate to tau= \\eta du/dy, which for a 5 cm radius flask makes the shear stress ~0.01 Pa, or 1/3 of the stress experienced by a swimming cell. Let's leave that for the journal clubs to discuss though - the authors' argument is reasonable.

- I still think that the disctinction that the authors make between 'velocity' and 'speed' is unconventional, but the additional clarification describing how they define terms has helped. I was a bit confused by the statement "['average velocity'] is a more general quantification of motility than 'total displacement after 10 seconds,' which is dependent on the somewhat arbitrary choice to use 10 seconds of tracked data." The choice is a bit arbitrary, but that's what they're doing! Calling the quantity 'velocity' doesn't make it any less so, and presumably they would get different numbers if they used 5 or 20 seconds of data? At any rate, the definition is clear, so I'll leave it at that.

Reviewer #2: Line 143-161: The rationale for the FLAM3 RNAi belongs in the results, in context, not at the very beginning of the paper.

Lines 153, 155: It would be better to say that FLAM3 RNAi produces T. brucei cells with epimastigote-like morphologies. rather than referring to them as "leishmania-like". Leishmania also have forms that lack extended flagella, so this terminology is vague.

Line 258-292: Omit the data that does not pertain directly to LC2 RNAi only. Therefore, there is no clustering phenotype. Unless you can specify why FLAM3 RNAi would exacerbate LC2 RNAi?

Line 293: "basal body duplication followed by nuclear and kinetoplast DNA replication initiates cilium replication"- this statement is unclear.

Lines 300-308: LC2 RNAi on its own does not cause the appearance of aberrant DNA states. It is unclear what the authors are specifically talking about in Fig 3B- one image is just a DAPI channel- is this the RNAi case, if so, which is the RNAi? The fact that one kinetoplast is too near one nucleus in what appears to be a 4N2K cell is picking a small defect out of a cell with much more severe issues. I would omit this section because LC1 RNAi on its own does not appear to cause DNA defects.

Line 301-311: The 1N1K cell state does not mean that the cells are not dividing- they almost certainly are. It just means that they have not segregated their DNA yet. Other earlier processes are likely to be ongoing.

Lines 320-338: LC2 RNAi does not cause any changes in cilia length. FLAM3 KD and FLAM3-LC2 KD RNAi cilia are slightly shorter, but there was no difference between them. Since there's no LC2-related phenotype, omit. TbCL2 RNAi does not disrupt cell division. Can be omitted.

Line 381: The difference in growth rates between control and LC2 RNAi cells is very small. I would be very cautious in claiming a difference in growth rate this small.

Line 393-412: Can be omitted.

Line 683-685: You cannot attribute these phenotypes to LC2 depletion alone- it's the synthetic effect of FLAM3 and LC2 depletion.

Generally, the Discussion barely mentions the cell division and morphology data prior to line 449, which strongly calls for discarding many parts of it and focusing the motility data.

PLOS authors have the option to publish the peer review history of their article (what does this mean?). If published, this will include your full peer review and any attached files.

Reviewer #1: No

Reviewer #2: No

Figure Files:

Data Requirements:

Reproducibility:

References:

---

## [Editor Report · Decision Letter 2]

12 Jul 2022

Dear Dr. Alper,

Thank you very much for submitting your manuscript "Light chain 2 is a Tctex-type related axonemal dynein light chain that regulates directional ciliary motility in Trypanosoma brucei" for consideration at PLOS Pathogens. Your revised manuscript was reviewed by members of the editorial board. Based on the reviews, we are likely to accept this manuscript for publication, providing that you modify the manuscript according to the recommendations below.

Line 36: Remove “, including trypanosomes.”, as this is redundant.

Line 217:  Change text by deleting “semi-quantitiative”: Note that one can be quantitative with varied levels of precision, but “semi”-quantitative is not an appropriate description for quantitation with low precision. Text should read: “using reverse transcription PCR.”

Lines 286 – 294: Delete this entire paragraph, as per recommendation of reviewer 2. As noted previously, this is speculation. If needed, you can cite Fig S7 and text S1 at end of line 285.

Lines 302 – 310:  Delete this entire paragraph, as per recommendation of reviewer 2. As noted previously, this is speculation.

Lines 610 – 616:  Edit to remove the morphology and division, particularly exacerbation by FLAM3 – for reasons pointed out by reviewer 2. You lead with that comment in line … and add the more relevant and more strongly-supported results on ciliary motility analysis as an afterthought.  Edit to read as indicated below:

“We found that TbLC2 knockdown impaired cellular swimming (Fig 4B) and lowered directional swimming persistence (Fig 6). These cellular phenotypes were correlated with higher cilium beating frequencies (Fig 5), increased static (time-averaged) cilium curvature (Fig 7), greater dynamic cilium curvature asymmetries (Fig 7), and a shift in beat mode bias away from the forward motility tip-to-base bending wave propagation mode (Fig 7).”

Lines 736 – 759:  Delete this entire section, because it is too speculative and detracts from the important and well-supported work on impact of LC2 on ciliary beating, as pointed out in prior reviews.

Sincerely,

Kent L. Hill

Associate Editor

PLOS Pathogens

David Sacks

Section Editor

PLOS Pathogens

Kasturi Haldar

Editor-in-Chief

PLOS Pathogens

orcid.org/0000-0001-5065-158X

Michael Malim

Editor-in-Chief

PLOS Pathogens

orcid.org/0000-0002-7699-2064

Reviewer Comments (if any, and for reference):

Figure Files:

Data Requirements:

Reproducibility:

References:

---

## [Editor Report · Decision Letter 3]

26 Aug 2022

Dear Dr. Alper,

We are pleased to inform you that your manuscript 'Light chain 2 is a Tctex-type related axonemal dynein light chain that regulates directional ciliary motility in Trypanosoma brucei' has been provisionally accepted for publication in PLOS Pathogens.

Best regards,

Kent L. Hill

Associate Editor

PLOS Pathogens

David Sacks

Section Editor

PLOS Pathogens

Kasturi Haldar

Editor-in-Chief

PLOS Pathogens

orcid.org/0000-0001-5065-158X

Michael Malim

Editor-in-Chief

PLOS Pathogens

orcid.org/0000-0002-7699-2064
---

## [Editor Report · Acceptance letter]

15 Sep 2022

Dear Dr. Alper,

We are delighted to inform you that your manuscript, "Light chain 2 is a Tctex-type related axonemal dynein light chain that regulates directional ciliary motility in Trypanosoma brucei," has been formally accepted for publication in PLOS Pathogens.

Best regards,

Kasturi Haldar

Editor-in-Chief

PLOS Pathogens

orcid.org/0000-0001-5065-158X

Michael Malim

Editor-in-Chief

PLOS Pathogens

orcid.org/0000-0002-7699-2064