# Supplementary material for: Light chain 2 is a Tctex-type related axonemal dynein light chain that regulates directional ciliary motility in Trypanosoma brucei
Source: PLoS Pathog. 2022 Sep 26;18(9):e1009984. doi: 10.1371/journal.ppat.1009984 (PMC9536576; doi:10.1371/journal.ppat.1009984)
Supplement: S1 Text — (PDF) [file ppat.1009984.s001.pdf]

Supporting information for "Light chain 2 is a Tctex-type related axonemal dynein light chain that regulates directional ciliary motility in *Trypanosoma brucei*"

Subash Godar[1,2], James Oristian[2,3,#a], Valerie Hinsch[2,3,#b], Katherine Wentworth[2,4], Ethan Lopez[2,3], Parastoo Amlashi[2,4,#c], Gerald Enverso[2,4], Samantha Markley[2,4], and Joshua Alper[1,2,4,#d]

1 Department of Physics and Astronomy, College of Science, Clemson University, Clemson, SC 29634, United States of America
2 Eukaryotic Pathogens Innovation Center, Clemson University, Clemson, SC 29634, United States of America
3 Department of Genetics and Biochemistry, College of Science, Clemson University, Clemson, SC 29634, United States of America
4 Department of Biological Sciences, College of Science, Clemson University, Clemson, SC 29634, United States of America
#a Current Address: Department of Infectious Disease and the Center for Tropical and Emerging Global Disease, University of Georgia, Athens, GA 30602, United States of America
#b Current Address: Department of Biological Sciences, University of South Carolina, Columbia, SC 29208, United States of America
#c Current Address: Koch Institute of Integrative Cancer Research, Massachusetts Institute of Technology, Cambridge, MA 02142, United States of America
#d Current Address: GlaxoSmithKline, Cambridge, MA 02140, United States of America

Supporting Results and Discussion

TbLC2 knockdown causes morphological phenotypes associated with incomplete cytokinesis

We examined the effect of LC2 knockdown on the morphology of cultured trypanosome cells using wide-field microscopy (Supplementary Materials and Methods). We observed FLAM3-LC2 KD cells forming clusters consisting of two to over ten cells after 72 hours of RNAi induction (S7A Fig, *top*), while uninduced, FLAM3 KD (S7A Fig, *top*), LC2 KD, and WT/LC2 OE (S14 Fig) cells did not. We also observed FLAM3-LC2 KD/LC2 OE cells forming fewer clusters than FLAM3-LC2 KD cells (S7A Fig, *top*). These observations suggest that LC2 knockdown leads to a FLAM3 knockdown amplified cell division phenotype.

Several *T. brucei* cell lines with cytokinesis-based cell division defects, including PFR2[1,2], dynein intermediate chain 138 (IC138)[3], dynein light chain 1 (LC1)[4], and trypanin[5] knockdown cells, exhibit correlated motility phenotypes. Because TbLC2 knockdown likely caused a cell division phenotype (S7A Fig, *top*) and LC2 depletion causes ciliary motility defects[6,7] in *C. reinhardtii*, we hypothesized that cell motility-like fluid shear forces (see order of magnitude estimate on the shear stresses on trypanosome cells, below, showing that shaking exerts fluid shear stress[8] on the trypanosome cells that are one half to one order of magnitude larger than the fluid shear stresses exerted by ciliary based swimming motility[9,10]) induced by shaking the cell cultures might rescue TbLC2 knockdown cilium motility-based cell division phenotypes. Therefore, we compared the propensity of the cells to form clusters in various cell lines when cultured with and without shaking (Supplementary Materials and Methods). We found that gentle shaking (80-90 rpm) resulted in somewhat fewer and smaller cell clusters than no shaking in FLAM3-LC2 KD and essentially eliminated clustering in FLAM3-LC2 KD/LC2 OE cells (S7A Fig, *bottom*). These observations suggest that the TbLC2 knockdown cell division phenotype may be due, in part, to a motile cilium-based cytokinesis defect. However, we only found a slight, but not significant, increase in growth rate of FLAM3-LC2 KD cells with shaking after 72 hours (p-value = 0.9, two-tailed paired t-test, S8 Fig). This result was consistent with the observation that shaking only minimally reduced cel cluster formation (S7A Fig) and had no effect on cell clump formation (S7D Fig). Together, these results suggest TbLC2 knockdown may cause an additional, possibly indirect or ciliary beating waveform-related, effect on the cytokinesis phenotype because external shear force could not rescue the cell separation phenotypes[5] as was predicted using a reaction force model[11].

Replication of its cilium is an essential part of *T. brucei*'s cell cycle[12]. Basal body duplication followed by nuclear and kinetoplast DNA replication precedes cilium replication[13]. Therefore, abnormalities in cell cycle progression frequently result in nucleus and kinetoplast number and localization defects characterized by kinetoplasts being close to or sometimes indistinguishable from the nuclei[3].

We imaged DAPI-stained trypanosome cells to characterize the number and relative separation of the nuclei and kinetoplasts in multiple LC2 knockdown and control cell lines (S7B Fig, Supplementary Materials and Methods). We found that LC2 knockdown cells were more likely to have kinetoplasts closer to the nucleus than expected (1/3rd or less than the average separation in LC2 KD and nearly adjoining in FLAM3-LC2 KD cells, S7B Fig), as compared to their respective parent cell lines (S7C Fig). We categorized the cells according to the number and localization of kinetoplasts and nuclei observed in cells (xK yN, S7B Fig). We found the percentage of cells with one nucleus well-separated from one kinetoplast (1N 1K, S7B Fig), was 1.3-fold reduced in LC2 KD cells (p-value < 0.001, two-tailed paired t-test, S7C Fig), as compared to uninduced cells. However, the multi-kinetoplast multi-nucleus (MK MN, S7B Fig) mislocalization classification, which correlates to multi-ciliated amorphous cellular clumps unable to undergo multiple cycles of complete cytokinesis (S7D Fig), was entirely absent from the populations of both uninduced and LC2 KD cells. Together, these observations suggest that the knockdown of TbLC2 in wild-type cells may slow cell division, as there are fewer 1K 1N cells, a classification associated with cells not currently undergoing cell division. However, these data suggest that LC2 KD cells undergo normal cytokinesis, as there are no instances of the MK MN classification in these cells (S7C Fig).

We also found that FLAM3 KD cells exhibited a 2.3-fold reduction in 1K 1N cells, as compared to uninduced cells (p-values < 0.0001, two-tailed paired t-test, S7C Fig) and a small (4.1%), though significant (p-value = 0.026, two-tailed single sample t-test, S7C Fig), fraction of MK MN cells. Knockdown of TbLC2 in FLAM3 cells (FLAM3-LC2 KD cells) further reduced the fraction of 1K 1N (13.5-fold reduced, p-value < 0.0001, two-tailed paired t-tests, S7C Fig), as compared to uninduced cells. Moreover, we observed that the MK MN classification occurred at significantly higher frequency in the FLAM3-LC2 KD than in FLAM3 KD cells (4.7-fold, p-value < 0.0001, two-tailed paired t-test, S7C Fig).

Cytokinesis defects in *T. brucei* often correlate either to defects in the length of the cilium or to the biophysical properties of the ciliary beat[5,14]. Therefore, we compared the lengths of wild type cilia extracted using biochemical cell fractionation (Supplementary Materials and Methods) and FLAM3 KD and FLAM3-LC2 KD cilia extracted using mechanical shearing (Supplementary Materials and Methods). We found that the wild-type cilia were 18.3 ± 0.4 µm (mean ± SEM, N = 76), FLAM3 KD cilia were 16.8 ± 0.4 µm (N = 76), and FLAM3-LC2 KD cilia were 17.1 ± 0.3 µm (N = 52) long (S6 Fig). The length of FLAM3 KD and FLAM3-LC2 KD cilia were not significantly different (p-value = 0.64, two-tailed paired t-tests, S7 Fig), suggesting TbLC2 knockdown does not affect the length of the cilium. However, we found that FLAM3 KD and FLAM3-LC2 KD cilia measured slightly (< 10%), but significantly (p-values = 0.007 and 0.02, respectively, two-tailed paired t-tests), shorter than wild-type cells (S6 Fig), either due to the differences in cilia extraction method or the effects of FLAM3 knockdown[15].

We extended the analysis on WT/LC2 OE cells to investigate any possible effect of overexpression on cell morphologies associated with cytokinesis defect. We found that the WT/LC2 OE cell line showed a 2.2-fold decrease in the fraction of 1K 1N cells as compared to wild-type (p-value < 0.0001, two-tailed paired t-test, S7C Fig), and it showed a non-zero fraction of the cells exhibiting the mislocalized multi-kinetoplast, multi-nucleus phenotype (MK MN, p-value = 0.003, two-tailed t-test, S7C Fig), as opposed to uninduced cells, which did not exhibit the MK MN phenotype at all (S7C Fig).

Together, these observations suggest that that there is an optimal level of TbLC2 expression in the cell and knocking it down disrupts cell division at cytokinesis after undergoing ciliary neogenesis. Because the TbLC2 only knockdown in LC2 KD cells did not lead to new FAZ formation defects (as evidenced by absence of detached flagellum, Fig 2C, *left*), we expect the TbLC2 knockdown in the FLAM3-LC2 KD cells to not further affect the FAZ formation. Additionally, since gentle shaking of the cell cultures partially rescues this phenotype, these observations further suggest that the TbLC2 knockdown-related cytokinesis phenotype may be due to ciliary motility defects. Moreover, because TbLC2

knockdown does not strongly affect the length of the cilium, the results suggest that TbLC2 regulates the beating waveform of trypanosome cilia.

The effect of TbLC2 knockdown on cell motility is more pronounced in FLAM3 KD than in wild-type parental cells

We found that TbLC2 RNAi knockdown in both wild-type (LC2 KD) and FLAM3 knockdown (FLAM3-LC2 KD) cells showed significant microscopic cell motility phenotypes, with the effect being more severe in the FLAM3 knockdown background. To better understand how FLAM3 knockdown caused the amplified TbLC2 phenotypes that we observed, we consider that stiffer, morphologically straighter trypanosome cells swim with straighter trajectories than flexible cells.[16] Additionally, the directional persistence of *Spiroplasma*, a helical-shaped flexible bacterium without cell walls that swims in helical paths similar to *T. brucei*, suggests that less rigid cells are more susceptible to environmental perturbations, leading to a reduction in their swimming directional persistence.[17] The subpellicular microtubule corset,[18] which lies directly under *T. brucei*'s plasma membrane and is organized by the FAZ,[19] provides structural integrity and rigidity to the trypanosome's cell body.[20,21] When the cilium remains attached to the cell body by the FAZ, the cell body-subpellicular microtubule array increases the effective flexural rigidity of the cilium by more than the simple arithmetic sum of the cilium and cell body stiffnesses. When the cilium becomes detached, it loses this synergy and effectively becomes much softer. Moreover, the cilium's attachment to its cell body induces the chirality that causes trypanosomes to rotate about their long cell body axis, which is a dominant contributor to their swimming directional persistence.[22] Although the ciliary beating waveform of FLAM3 knockdown cells maintains its helicity, perhaps because the paraflagellar rod remains attached to the cilium (Fig 2), FLAM3 knockdown cells exhibit a significant reduction in cell body rotation.[22,23]

The combined effects of increased stiffness and cell swimming long-axis rotation, both of which enhance trypanosome swimming motility, get neutralized when trypanosome cells have a detached cilium. Thus, the effects of genetic (e.g., loss of function mutants) and environmental (e.g., increased viscosity) perturbations that impair the ciliary beat become more pronounced in trypanosome cells with a detached cilium, as compared to their effects on trypanosome cells with intact FAZs.[24] Together, all this evidence suggests that the biophysical consequences of dissociating the cilium-cell body complex combine to amplify the motility phenotypes of TbLC2 knockdowns in FLAM3 knockdown cell lines, as compared to TbLC2 knockdowns in wildtype cells.

Order of magnitude estimate on the shear stresses on trypanosome cells

We performed an order of magnitude analyses on the fluid shear forces acting on a swimming trypanosome cell and during mechanical agitation of a culture flask.

We assumed that the shear stress due to Stokes drag (low Reynolds number) on the cell scales like $\tau_{\text{swim}} \quad \frac{3}{2}\frac{\eta U}{R}$, where η is the viscosity of the culture medium ($\sim 0.001 \frac{\text{pN s}}{\mu\text{m}^2}$), U is the swimming speed of trypanosome cells (~ 20 $\frac{\mu\text{m}}{\text{s}}$), and R is the characteristic radius of a trypanosome cell (~ 1 μm). We found that the estimated shear stress on a swimming trypanosome cell was $\tau_{\text{swim}} \quad 0.03 \frac{\text{pN}}{\mu\text{m}^2}$.

We assumed that the shear stress in the culture flask being subjected to orbital shaker oscillation was $\tau_{\text{flask}} \quad a\sqrt{\eta\rho\left(2\pi f\right)^3}$, where a is the orbital radius of rotation of the shaker (~ 1 cm), ρ is the density of the culture medium (~ 1 g/mL), and *f* is the frequency of orbital shaker rotation (~ 1.5 Hz). We found that the estimated shear stress on a trypanosome cell in culture on an orbital shaker was $\tau_{\text{flask}} \quad 0.3 \frac{\text{pN}}{\mu\text{m}^2}$.

Supporting Tables

Table A. Primer sets used in PCR amplifications and for Sanger sequencing. The base pairs in lower case represent overlap regions with adjacent DNA fragments in an assembly. Highlighted base pairs represent restriction sites engineered into the primer sets.

Primer name	Sequence
TbLC2 Forward	5'-attacaccaaaaagtagatcggtatCGATTGTATGCAAACAGATTTTG-3'
TbLC2 Reverse	5'-tccctcgtaggcgCGCCCATAATATTGATAGAG-3'
His6/eGFP Forward	5'- catcaagccaaagCTTCACCACCACCACCAC-3'
His6/eGFP Reverse	5'- gctttacacgcttgcatccgcttagAATTCTTACTTGTACAGCTCGTC-3'
BCCP Forward	5'- aatattatgggcgCGCCTACGAGGGATCCGC-3'
BCCP Reverse	5'- ggtggtggtggtgaagCTTTGGCTTGATGATCATGATGGGC-3'
TbLC2.BCCP.His6/eGFP Forward	5'- accaaaaagtaaaattcacaagcttATGCAAACAGATTTTGGAG-3'
TbLC2.BCCP.His6/eGFP Reverse	5'-aaagccaactaaatgggcaggatccTTACTTGTACAGCTCGTC-3'
pLEW seq Forward	5'- CTGCACGCGCCTTCGAGT-3'
pLEW seq Reverse	5'- ACCTCCCTGCTGTGCCAT-3'
FLAM3 Forward	5'-CCGCTCGAGAAGATCGTTGGAGGTACCAAGTG-3'
FLAM3 Reverse	5'-CCCAAGCTTGCGACAGCGTTCAGAGGC-3'
TbLC2 3'-UTR XH Forward	5'- ACCTATCTCGAGCAACTCTTCCACCGTGTCTATTCG-3'
TbLC2 3'-UTR XH Reverse	5'- ACCTATAAGCTTCACGGATAATCTCCTACCCATGGAA-3'
TbLC2 3'-UTR XX Forward	5'- ACCTATCTCGAGCAACTCTTCCACCGTGTCTATTCG-3'
TbLC2 3'-UTR XX Reverse	5'- ACCTATCTCGAGACACGGATAATCTCCTACCCATGGA-3'

Table B. Cell lines generated and used in the study. The gene contents on all the cells listed below (except WT) are based on the doxycycline induced state.

Cell line name	Plasmid (S11 Fig for details)	FLAM3	Endogenous TbLC2	Recombinant TbLC2
WT	NA	Yes	Yes	No
LC2 KD	pZJM.TbLC2 RNAi	Yes	No (reduced)	No
FLAM3 KD	pZJM.FLAM3 RNAi	No (highly reduced)	Yes	No
FLAM3-LC2 KD	pZJM.FLAM3.TbLC2 RNAi	No (highly reduced)	No (reduced)	No
FLAM3-LC2 KD/ LC2 OE	pZJM.FLAM3.TbLC2 RNAi and pLEW.TbLC2::BCCP::eGFP/His6	No (highly reduced)	No (reduced)	Yes
WT/LC2 OE	pLEW.TbLC2::BCCP::eGFP/His6	Yes	Yes	Yes

Supplementary materials and methods

Nuclei and kinetoplast characterization

We quantified the number and separation of nuclei and kinetoplasts in cell various cell populations to characterize the defects in the replication and translocation of nuclear and kinetoplast DNA during the cell division. We fixed the cells, stained their DNA using DAPI containing mounting solution (DAPI Fluoromount-G, 0100-20, Southern Biotech, Birmingham, AL), and imaged them using a wide-field fluorescence microscope, as described above. We classified the cells as xKyN, where x is the number of kinetoplasts and y is the number of nuclei present, and x and y = N indicates cells with more than two nuclei and kinetoplasts as previously described[3,25].

Extraction of cilia

We harvested 1×10^8 FLAM3 RNAi-induced cells by centrifuging at 1500×g for 10 min at 4°C, washed the pellets in PEME buffer (EGTA 2 mM, $MgSO_4$ 1 mM, EDTA 0.1 mM, PIPES free acid 0.1 mM, pH 6.9), and resuspend them into a 50 mL conical tube with 2 mL of PEME. We vortexed the cells for 15 minutes at 3200 rpm and centrifuged them at 420×g in a swinging bucket rotor for 10 minutes. We separated the dissociated cilia from the cell bodies by transferring the supernatant to 1.5 mL microcentrifuge tubes and centrifuging them at 420×g for 5 minutes. We saved the supernatant (cilia extract) and resuspended all the pellets in a 50 mL conical tube with 2 mL of PEME buffer. We performed a second separation by repeating the centrifugation steps. We combined the supernatant-containing ciliary extracts and centrifuged them at 25,000×g for 20 minutes. We stored the cell body and ciliary pellets at -80°C.

We modified a previously published method of cell fractionation to extract the cilia[26] from FLAM3 uninduced or wild-type cells with cilia still intact to the cell body. Briefly, we first harvested and washed 5×10^7 cells with PEME buffer as described above. We then detergent extracted the cells by resuspending the cell pellet in PEME buffer with 1% IGEPAL (CA-630, Alfa Aesar, MA) and incubating on ice for 15 minutes. We centrifuged the cells at 3400×g for 6 minutes at 4°C and saved the membrane and cytoplasmic content-containing supernatant (S1) and the cilia and subpellicular microtubule corset-containing pellet (P1). We resuspended P1 in PEME buffer with 1% IGEPAL and 1 M NaCl and incubated the mixture for 45 minutes on ice to depolymerize the corset microtubules. We then centrifuged the mixture at 16000×g for 15 minutes at 4°C to separate it into a depolymerized corset microtubule and associated protein-containing supernatant (S2) and an axoneme, paraflagellar rod, and basal body-containing pellet (P2).

Supplementary References

1. Portman, N. & Gull, K. The paraflagellar rod of kinetoplastid parasites: From structure to components and function. *Int J Parasitol* **40**, 135–148 (2010).
2. Lander, N., Li, Z.-H., Niyogi, S. & Docampo, R. CRISPR/Cas9-Induced Disruption of Paraflagellar Rod Protein 1 and 2 Genes in Trypanosoma cruzi Reveals Their Role in Flagellar Attachment. *mBio* **6**, e01012-15 (2015).
3. Marchese, A., Wilson, C. & Springer, A. L. Knockdown of axonemal inner arm dynein protein IC138 Trypanosoma brucei causes flagellar detachment and defects in motility. *The FASEB Journal* **30**, 1067.2-1067.2 (2016).
4. Baron, D. M., Kabututu, Z. P. & Hill, K. L. Stuck in reverse: loss of LC1 in Trypanosoma brucei disrupts outer dynein arms and leads to reverse flagellar beat and backward movement. *J Cell Sci* **120**, 1513–1520 (2007).
5. Ralston, K. S., Lerner, A. G., Diener, D. R. & Hill, K. L. Flagellar Motility Contributes to Cytokinesis in Trypanosoma brucei and Is Modulated by an Evolutionarily Conserved Dynein Regulatory System. *Eukaryot Cell* **5**, 696–711 (2006).
6. Pazour, G. J. *et al.* LC2, the Chlamydomonas Homologue of the t Complex-encoded Protein Tctex2, Is Essential for Outer Dynein Arm Assembly. *Mol Biol Cell* **10**, 3507–3520 (1999).
7. DiBella, L. M. *et al.* The Tctex1/Tctex2 Class of Dynein Light Chains: DIMERIZATION, DIFFERENTIAL EXPRESSION, AND INTERACTION WITH THE LC8 PROTEIN FAMILY. *J Biol Chem* **276**, 14366–14373 (2001).
8. Warboys, C. M., Ghim, M. & Weinberg, P. D. Understanding mechanobiology in cultured endothelium: A review of the orbital shaker method. *Atherosclerosis* **285**, 170–177 (2019).
9. Stellamanns, E. *et al.* Optical trapping reveals propulsion forces, power generation and motility efficiency of the unicellular parasites Trypanosoma brucei brucei. *Scientific Reports* **4**, 6515 (2014).
10. Dardik, A. *et al.* Differential effects of orbital and laminar shear stress on endothelial cells. *Journal of Vascular Surgery* **41**, 869–880 (2005).
11. Absalon, S. *et al.* Basal Body Positioning Is Controlled by Flagellum Formation in Trypanosoma brucei. *PLOS ONE* **2**, e437 (2007).

12. Dutta, S. & Avasthi, P. Flagellar Synchronization Is a Simple Alternative to Cell Cycle Synchronization for Ciliary and Flagellar Studies. *mSphere* **2**, e00003-17 (2017).
13. Vaughan, S. & Gull, K. Basal body structure and cell cycle-dependent biogenesis in Trypanosoma brucei. *Cilia* **5**, 5 (2016).
14. Kohl, L., Robinson, D. & Bastin, P. Novel roles for the flagellum in cell morphogenesis and cytokinesis of trypanosomes. *EMBO J* **22**, 5336–5346 (2003).
15. Zhou, Q., Liu, B., Sun, Y. & He, C. Y. A coiled-coil- and C2-domain-containing protein is required for FAZ assembly and cell morphology in Trypanosoma brucei. *Journal of Cell Science* **124**, 3848–3858 (2011).
16. Uppaluri, S. *et al.* Impact of Microscopic Motility on the Swimming Behavior of Parasites: Straighter Trypanosomes are More Directional. *PLOS Computational Biology* **7**, e1002058 (2011).
17. Wada, H. & Netz, R. R. Hydrodynamics of helical-shaped bacterial motility. *Phys. Rev. E* **80**, 021921 (2009).
18. Angelopoulos, E. Pellicular Microtubules in the Family Trypanosomatidae. *The Journal of Protozoology* **17**, 39–51 (1970).
19. Gull, K. The Cytoskeleton of Trypanosomatid Parasites. *Annu. Rev. Microbiol.* **53**, 629–655 (1999).
20. Vedrenne, C. *et al.* Two Related Subpellicular Cytoskeleton-associated Proteins inTrypanosoma brucei Stabilize Microtubules. *MBoC* **13**, 1058–1070 (2002).
21. Sinclair, A. N. & Graffenried, C. L. de. More than Microtubules: The Structure and Function of the Subpellicular Array in Trypanosomatids. *Trends in Parasitology* **35**, 760–777 (2019).
22. Wheeler, R. J. Use of chiral cell shape to ensure highly directional swimming in trypanosomes. *PLoS Comput Biol* **13**, e1005353 (2017).
23. Sun, S. Y. *et al.* Flagellum couples cell shape to motility in Trypanosoma brucei. *PNAS* **115**, E5916–E5925 (2018).
24. Constantino, M. A., Jabbarzadeh, M., Fu, H. C. & Bansil, R. Helical and rod-shaped bacteria swim in helical trajectories with little additional propulsion from helical shape. *Science Advances* **2**, e1601661 (2016).

25. Selvapandiyan, A., Kumar, P., Salisbury, J. L., Wang, C. C. & Nakhasi, H. L. Role of Centrins 2 and 3 in Organelle Segregation and Cytokinesis in Trypanosoma brucei. *PLOS ONE* **7**, e45288 (2012).
26. Wei, Y., Hu, H., Lun, Z.-R. & Li, Z. Centrin3 in trypanosomes maintains the stability of a flagellar inner-arm dynein for cell motility. *Nat Commun* **5**, 4060 (2014).
