# Supplementary figures and images for: Light chain 2 is a Tctex-type related axonemal dynein light chain that regulates directional ciliary motility in Trypanosoma brucei
Source: PLoS Pathog. 2022 Sep 26;18(9):e1009984. doi: 10.1371/journal.ppat.1009984 (PMC9536576; doi:10.1371/journal.ppat.1009984)

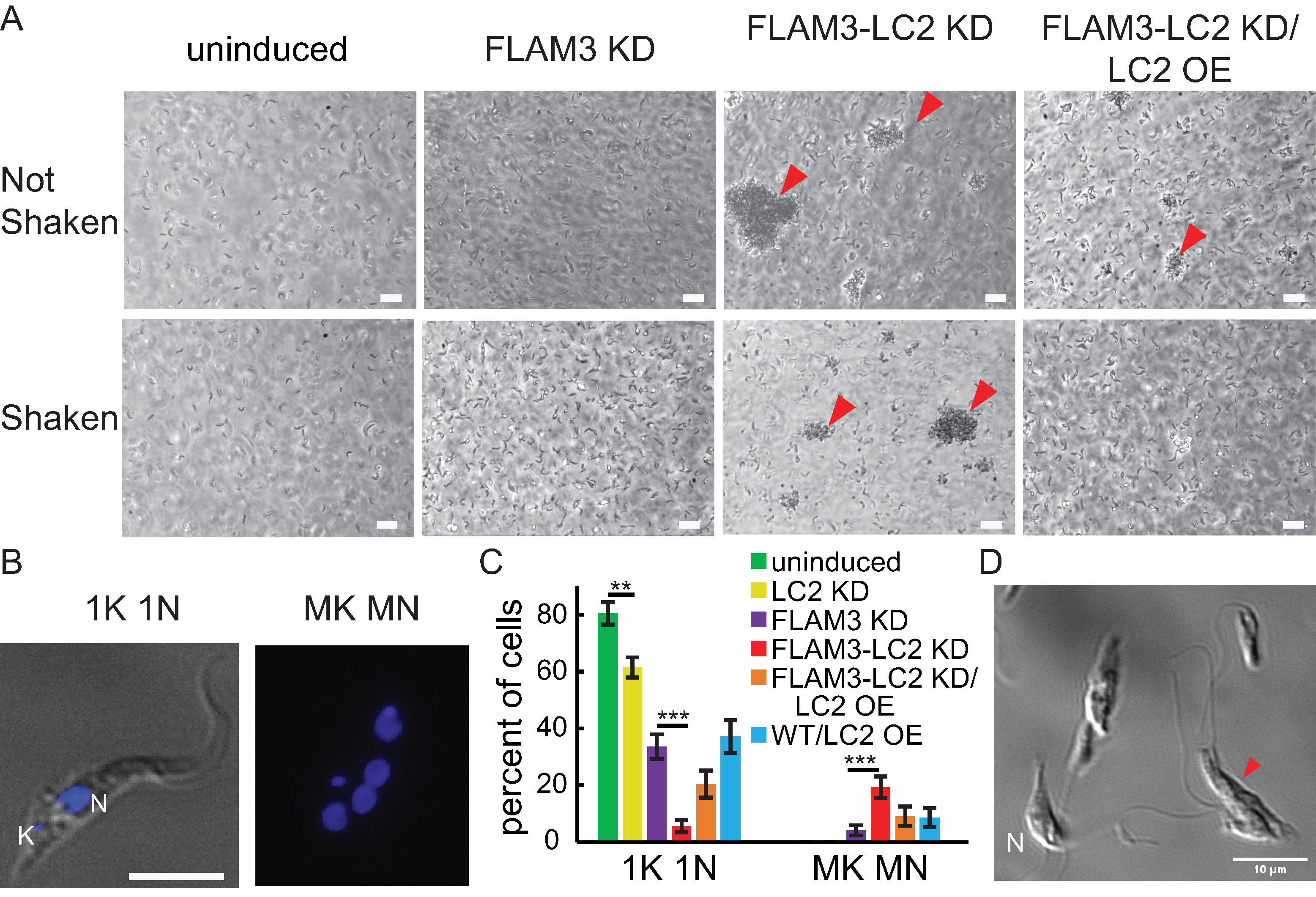

Supplement: S7 Fig — A. Representative images of uninduced, FLAM3 KD, FLAM3-LC2 KD, and FLAM3-LC2 KD/LC2 OE cells cultures in the culture flask using phase-contrast microscopy 72 hours post-induction when we did not shake (top) and shook (bottom) the flasks. Major clusters of cells are indicated (red arrows). The scale bars represent 10 μm. B. Representative DAPI stained images for classification of cells as having x kinetoplasts (xK) and y nuclei (yN). 1K 1N refers to cells with one kinetoplast normally localized to one nucleus (left). MK MN refers to cells classified as having multiple (M>2) mislocalized (closer to each other) kinetoplasts and nuclei (right), likely resulting from incomplete kinetoplast migration and/or incomplete cytokinesis. The scale bar represents 5 μm and both images in this panel have the same scale. C. Occurrence frequency of one kinetoplast and one nucleus, normally localized within the cell (1K 1N) and the occurrence frequency of the multi-kinetoplast, multi-nucleus (MK MN) classification, as described in panel B., in uninduced and induced (72 hours post-induction) LC2 KD, FLAM3 KD, FLAM3-LC2 KD, FLAM3-LC2 KD/LC2 OE, and WT/LC2 OE cells. N = 101, 192, 122, 111, 72, and 70 total classified cells of each strain, respectively. Other classifications, e.g., 1K 2N, 2K 1N, and 2K 2N, which likely include cells undergoing cell division, account for the percentages not represented. The error bars represent the statistical counting error. ** = p-value < 0.001 and *** = p-value < 0.0001, two-tailed paired t-tests. D. DIC microscopy image of fixed FLAM3-LC2 KD cells, including a representative amorphous clump of cells with multiple detached cilia (red arrow). (TIF) [file ppat.1009984.s008.tif]
