# Supplementary material for: Light chain 2 is a Tctex-type related axonemal dynein light chain that regulates directional ciliary motility in Trypanosoma brucei
Source: PLoS Pathog. 2022 Sep 26;18(9):e1009984. doi: 10.1371/journal.ppat.1009984 (PMC9536576; doi:10.1371/journal.ppat.1009984)
Supplement: Attachment — Submitted filename: Response to reviewers letter - 220814.pdf [file ppat.1009984.s030.pdf]

Dear PLOS Pathogens editors,

Thank you for these reviews and all the help you have provided throughout the submission process for this manuscript. Please see see ours responses to all review comments, and a description of the changes we have made in the manuscript, below, in blue.

Thank you,

Joshua Alper

Thank you very much for submitting your manuscript "Light chain 2 is a Tctex-type related axonemal dynein light chain that regulates directional ciliary motility in Trypanosoma brucei" for consideration at PLOS Pathogens. Your revised manuscript was reviewed by members of the editorial board. Based on the reviews, we are likely to accept this manuscript for publication, providing that you modify the manuscript according to the recommendations below.

We thank the editors for their time. We have incorporated the changes to the manuscript required by the editors. We have highlighted the changes we made in the manuscript and formulated our replies to each issue in blue below.

Line 36: Remove ", including trypanosomes.", as this is redundant.

We agree with the review and removed "including trypanosomes" from the text to correct the redundancy.

Line 217: Change text by deleting "semi-quantitiative": Note that one can be quantitative with varied levels of precision, but "semi"-quantitative is not an appropriate description for quantitation with low precision. Text should read: "using reverse transcription PCR."

We removed the term "semi-quantitative" from the text.

Lines 286 – 294: Delete this entire paragraph, as per recommendation of reviewer 2. As noted previously, this is speculation. If needed, you can cite Fig S7 and text S1 at end of line 285.

We removed this section of the manuscript but cited Fig S7 and text S1 at end of line 285, as suggested in the review.

Lines 302 – 310: Delete this entire paragraph, as per recommendation of reviewer 2. As noted previously, this is speculation.

We removed this paragraph, per the request from the editors.

Lines 610 – 616: Edit to remove the morphology and division, particularly exacerbation by FLAM3 – for reasons pointed out by reviewer 2. You lead with that comment in line … and add

the more relevant and more strongly supported results on ciliary motility analysis as an afterthought. Edit to read as indicated below:

"We found that TbLC2 knockdown impaired cellular swimming (Fig 4B) and lowered directional swimming persistence (Fig 6). These cellular phenotypes were correlated with higher cilium beating frequencies (Fig 5), increased static (time-averaged) cilium curvature (Fig 7), greater dynamic cilium curvature asymmetries (Fig 7), and a shift in beat mode bias away from the forward motility tip-to-base bending wave propagation mode (Fig 7)."

We modified this section as suggested by the editors.

Lines 736 – 759: Delete this entire section, because it is too speculative and detracts from the important and well-supported work on impact of LC2 on ciliary beating, as pointed out in prior reviews.

We removed this section from the main text, as suggested by the editors.
