# Supplementary material for: Light chain 2 is a Tctex-type related axonemal dynein light chain that regulates directional ciliary motility in Trypanosoma brucei
Source: PLoS Pathog. 2022 Sep 26;18(9):e1009984. doi: 10.1371/journal.ppat.1009984 (PMC9536576; doi:10.1371/journal.ppat.1009984)
Supplement: Attachment — Submitted filename: Godar et al. Repsonse to reviewers.docx [file ppat.1009984.s028.docx]

**Part I - Summary**
Please use this section to discuss strengths/weaknesses of study, novelty/significance, general execution and scholarship.

Reviewer #1: This paper describes the structure and function of a light-chain dynein-related protein in Trypanosoma brucei that the authors refer to as TbLC2. The authors investigate its localization and model its structure with a variety of standard methods. The effect of TbLC2 on motility is described at some length, in a series of experiments in which the authors use FLAM3 knockdown cells to enable better visualization of flagellar waveforms. The role of TbLC2 in regulating flagellar beating is discussed and compared to the role played by structurally similar proteins in other model microorganisms such as C. reinhardtii.

The authors begin by showing computational predictions of the structure of TbLC2, using some standard modelling frameworks – this is not my area of expertise, but the results seemed solid. Next the localization of the protein of interest was examined using fluorescence microscopy, and the authors have obtained some particularly convincing images of the adjacent localization of the paraflagellar rod (PFR) and LC2. The role of motility in assisting with the replicative cycle has been examined elsewhere, and the authors investigate this using shaken/unshaken cultures. They find that impairment of motility by LC2 is associated with larger cell clusters when the cultures are not shaken. Electron microscopy data shows that axoneme assembly is not contingent on TbLC2, unlike the analogous case in C. reinhardtii. Cells sediment more quickly in the case of the TbLC2 knockdown, even though the flagellar beat frequency is higher (measured by optical tweezers). The authors conduct a detailed video cell-tracking motility assay to determine the curvilinear speed and total displacement of the cells after ten seconds. Lastly, they perform a single-cell image analysis assay to investigate the asymmetry of the flagellar beat and fraction of ‘dwell time’ in either tip-to-base or base-to-tip propagation in the case of FLAM3 and LC2 knockdown.

This is a long and careful study, taking in many techniques and sets of experiments. Overall, I think that the paper is solid, and that the conclusions are justified by the results. The findings are novel (to my knowledge) and the execution is highly competent.

We thank the reviewer for the obviously careful reading of the manuscript.

I have a few fairly minor issues with the presentation of data, but my main complaint is that the manuscript is overlong, and repetitive in places. Some overlap between results/discussion/conclusion is to be expected (and I appreciate that this something of a matter of taste), but it’s tiring for the reader to encounter the same statements (e.g. those about flagellar asymmetry) in several places, in a similar context throughout a 56-page manuscript. I should also note that this only affects some parts - the optical tweezers section is succinct, for example - but the sections on the flagellar waveform in particular stuck out.

We appreciate that the initial manuscript is long and sometimes repetitive. We have attempted to address the redundancies in the revision. Specifically, we have eliminated duplications and redundant statements in the following sections:

1. We replaced much of the results summary / preview from the last paragraph of the introduction with a more detailed rational behind using the FLAM3 mutant (see discussion in response to other points, below).
2. We removed much of the details from the first paragraph of the discussion section that were redundant with the results.
3. We removed multiple paragraphs from the discussion section on flagellar waveforms. We moved some of the pertinent details from these paragraphs to the intro and results sections.
4. We consolidated multiple results subsections into a subsection centered on the primary conclusion of our work: that TbLC2 regulates trypanosome axonemal dynein
5. We merged much of the text on the effects of cell culture agitation with similar discussion in the results section.
6. Additionally, we made many other edits throughout the manuscript to make it more concise.

While the manuscript remains quite long, we hope that the reviewer agrees that the revised manuscript is both clearer and more succinct.

Reviewer #2: In this work, Godar and colleagues characterize a dynein light chain (LC2) in T. brucei using a variety of techniques, including loss-of-function experiments and live-cell imaging of laser-tweezer trapped cells to better understand the mechanics of flagellar beat in the absence of the protein. The manuscript as currently configured reads more like two separate papers (LC2 phenotypic characterization, flagellar beat studies) and because of this is quite long. There are a few places where the authors have broken from nomenclature conventions that make following the work confusing. For example, in my understanding the "delta" nomenclature the authors use to label their RNAi cell lines should only be used for true knockouts, where the gene itself has been removed from the cells, not just suppressed by RNAi. Also, there are multiple types of overexpression being used to modulate LC2- some tet-inducible, others not, that are not clearly detailed in the text.

We thank the reviewer for the obviously careful reading of the manuscript. We appreciate that the manuscript is long and covers a lot of material. We see the point about the manuscript reading like two separate papers. We have carefully considered ways we might split it into two manuscripts, but we were unable to arrive at a satisfying way to do so and create two, independent papers. Therefore, we have edited this single manuscript. We hope the reviewer sees the benefit of keeping the story together as one paper.

We agree that the nomenclature we used to label our cells was non-standard. Therefore, we have edited the names of the cell lines, eliminating the Δ nomenclature for knockdowns. The new nomenclature can be found in Table B of S1 Text in the supplementary materials. In summary, the principal cell lines we generated and used in this study are now named:

- WT
- LC2 KD
- FLAM3 KD
- FLAM3-LC2 KD
- FLAM3-LC2 KD/ LC2 OE
- WT/LC2 OE

We hope that this modified naming scheme adequately addresses the reviewer’s concern.

We thank the reviewer for highlighting the confusion about the overexpression lines used in this study. We have actually only used the pLEW100V5 tetracycline inducible expression plasmid system to overexpress proteins (TbLC2::eGFP) in trypanosome cells. We have edited the manuscript in multiple locations, including where TbLC2::eGFP overexpression is first mentioned and in the materials and methods, to clarify this point.

**Part II – Major Issues: Key Experiments Required for Acceptance**
Please use this section to detail the key new experiments or modifications of existing experiments that should be absolutely required to validate study conclusions.

Generally, there should be no more than 3 such required experiments or major modifications for a "Major Revision" recommendation. If more than 3 experiments are necessary to validate the study conclusions, then you are encouraged to recommend "Reject".

Reviewer #1: (No Response)

Reviewer #2: One of my biggest criticisms is that the authors do not really explain why they are doing essentially all their experiments in a FLAM3 RNAi background until the discussion. Much of the LC2 characterization is done without really regarding what the additional effect of FLAM3 depletion could be having on the cells when LC2 is perturbed. This needs to be clearly stated and accounted for. Is it not possible to do the biophysical analysis of the flagellar beat with an attached flagella?

We agree with the reviewer that the manuscript would greatly benefit from moving the rationale behind using the FLAM3 knockdowns to the introduction. Therefore, we moved some of the details we did not mention until the text around Fig 8 and the discussion section in the original manuscript to the introduction section. Most significantly, we replaced much of the results summary / preview from the last paragraph of the introduction with a more detailed rational behind using the FLAM3 mutant in the revised manuscript.

Additionally, we provide more detail on the rationale behind, additional effects of, and the consequences of using FLAM3 in each experiment, as we present them in the results section. We also changed the emphasis to highlight the effects of LC2 knockdown on wild-type cells in the results section before mentioning the effects of LC2 knockdown on FLAM3 knockdown cells, when the methods enabled a quantitative analysis. Specifically, we significantly modified and added to the text around Fig 2, S4 Fig, Fig 3 (added to supplement S13 Fig), Fig 4, Fig 5, Fig 7, and Fig 8.

The approach that the authors employ to "rescue" their LC2 RNAi produces a significant growth defect on its own, which makes it difficult to see it as a true rescue experiment. Even though the authors clearly show that it has a deleterious effect, they employ it as a control in their flagellar beat experiments. If overexpression is toxic, the authors could switch to an RNAi strategy where a portion of the coding sequence is recoded and then target the native sequence for RNAi. That would allow them to create an RNAi-insensitive allele at the native locus, which should have more wild-type expression levels.

We thank the reviewer for this thoughtful comment about the work. We agree that the “rescue” cells do not represent a true rescue. Therefore, we have edited the manuscript in many locations to eliminate the term “rescue” in reference to LC2 overexpressing cell lines and the partial restoration of wild-type phenotypes in these cell lines.

We appreciate that a true rescue could be generated by a number of strategies, including the strategy suggested by the reviewer, above, as well as CRISPR-Cas9 approaches, for example. However, after much consideration, we have decided not to pursue a true TbLC2 “rescue” cell line at this time. We made this decision because we do not think it would change any of the conclusions we drew with this work. We hope that the reviewers agree with us that such a cell line is not necessary, and that the edits we have made to the manuscript to remove references to “rescue” sufficiently describe the results and lead to logical conclusions, as they stand.

The degree of overexpression between the pXS2 O/E and the pLEW O/E should also be characterized by western blotting.
We apologize to the reviewer (and the readers) for the confusion introduced by the use of the pXS2 plasmid in the study. We never use pXS2 plasmid for anything other than a convenient intermediate vector from which to PCR amplify the assembled TbLC2.BCCP.His6/eGFP chimeric DNA in this work. We never use it to express LC2 in trypanosome cells in any of the data presented here. We clarified the extent to which we used the pXS2 plasmids in the materials and methods.

The phenotypic characterization of LC2 breaks with some of the best-practice approaches used in the field, such as the categorization of all non-1N1K cells as abnormal, instead of looking at the range of DNA states seen in a healthy, dividing cell population.

We thank the reviewer for pointing this issue out to us – the original text and Fig 3 caption was confusing. We edited the text and the caption of Fig 3 to clarify the presentation of the results and removed references to “severity” and “phenotype” of these classifications in an attempt to not correlate anything other than MK MN cells, which are associated with multi-ciliated amorphous cellular clumps (Fig 3D in revised manuscript), with abnormality. We clarified that other classifications like 2N 2K cells, for example, may be dividing, which is a perfectly normal process, in the caption to Fig 2, as suggested by the reviewer.

Also, the simplest analysis where just LC2 is depleted (rather than both FLAM3/LC2) should have been the starting point of the analysis, especially since the "rescue" cells where LC2 is likely to be significantly overexpressed are likely to have their own phenotypic issues.
We agree with reviewer’s comment and thank you for making this suggestion. In conjunction with Reviewer 2’s first two Major Issues, we reorganized the text describing the results in nearly every results subsection. We describe the Uninduced or WT vs LC2 result first, if possible. Only then did we introduce the results in the FLAM3 KD environment. Where appropriate, we emphasize that motility effects are likely be amplified by FLAM3, and we made sure to clarify these points in the discussion section titled “The effect of TbLC2 knockdown on cell morphology and motility is more pronounced in FLAM3 KD than in wild-type parental cells”. We also organized the results sections such that the LC2 overexpression results appear after the LC2 KD results, and are always qualified by language clearly denoting when phenotypes are partially or fully restored by LC2 overexpression. We hope that the reviewer agrees that the revised version of the manuscript is significantly clearer with respect to this point.

Considering that the flagellum is usually mostly attached in procyclic cells, it seems that this arrangement, which includes the FAZ and the transmission of the force from the flagellar beat into the MTs that surround the cell body, would have some effect on some of the parameters that the authors are measuring in their imaging. In some ways, this feels like a fairly artificial system for studying the function of LC2 because the cells have been perturbed already from their natural state by FLAM3 depletion. In other words, how do we know that LC2 function in an essentially detached flagellum reflects its function in an attached flagellum, which is its natural state in this form of the parasite?

To directly and briefly address the reviewer’s question: “how do we know that LC2 function in an essentially detached flagellum reflects its function in an attached flagellum, which is its natural state in this form of the parasite?”, we assume it does because there is no strong evidence to suggest that TbLC2’s axonemal dynein regulatory function would not reflect is function in an attached cilium. The ultrastructure of the axoneme, paraflagellar rod, and the flagellum as a whole is essentially unchanged by the FLAM3 KD, LC2 still localizes to the axoneme, and the direct interactions between LC2 and the dynein are unlikely to be changed by the FLAM3 KD. Therefore, we suggest that the FLAM3 KD is akin to altering the boundary conditions or the external loading on the axoneme, and that testing a dynamic biophysical system like the axoneme under multiple loading conditions sheds additional light on the underlying mechanisms of the system, rather than obscures those mechanisms. In this case, reducing the external loading on the cilium (by decoupling it from the subpellicular microtubule array) amplifies, rather than obscures, the role of TbLC2 on ciliary motility in trypanosomes.

See below for a more in-depth discussion:

This manuscript investigates the cell biological and biophysical role of TbLC2. We show that TbLC2 knockdowns in WT cells have cell division (Fig 3) and swimming motility phenotypes (Fig 7). We show that these phenotypes are likely related the way LC2 regulates the axonemal dynein-driven ciliary beating (e.g., the beat frequency Fig 6). As described above, we use the FLAM3 KD background to facilitate multiple quantitative characterizations of the beating ciliary waveforms. These quantitative characterizations enabled by FLAM3 KD augment the results we also present on TbLC2 KDs in WT cells. We added a paragraph at the end of the introduction section to clarify the rationale behind using FLAM3.

We absolutely agree with the reviewer that when the cilium is mostly attached to the cell body, as it is in wild-type procyclic trypanosome cells, the transmission of the force from the ciliary beat into the MTs that surround the cell body likely does have a significant effect on the parameters we measure. Additionally, the chiral nature of the attachment may play an important role, too. These ideas have been extensively addressed, in detail, in the literature. We included a detailed treatment of this in the discussion section of the manuscript – see that section for multiple references.

In our manuscript, we have shown that the effect of LC2 knockdown on ciliary motility, and hence the phenotypes related to the cell motility, is less severe in wild-type, as compared to FLAM3 KD cells. Therefore, because FLAM3 cells are more sensitive to the LC2 knockdown (similar to effects suggested by previous studies [Wheeler, PLOS Computational Biology 2017]), FLAM3 KD can made the effects of LC2 KD more pronounced, as we addressed in the discussion section, and clarified in the revised version, of the manuscript

Moreover, to better understand the molecular mechanisms of ciliary beat regulation more generally and to enable direct comparison other studies on cells with detached flagella, e.g., *Chlamydomonas* and *Leishmania*, we used FLAM3 knockdown cells. We have included these comparisons in the discussion section of the manuscript.

Finally, while the differences between the physical constraints on the cilium in wild-type and FLAM3 KD cells are likely to be significant, we argue that the underlying function of TbLC2 in the regulation of axonemal dynein is likely the same in both the WT and FLAM3 knockdown cells (e.g., the ultrastructure of the cilium is not altered by FLAM3 KD). Therefore, FLAM3 KD essentially provides a slightly altered set external loading conditions (as changing viscosity might have been) with which to test our hypotheses on the regulatory mechanisms of TbLC2. We have also included this idea in the discussion section of the manuscript.

For all these reasons, we believe that the use of FLAM3 KD is not only justified, but essential to deeply probing the function of TbLC2, despite the somewhat “artificial” nature of the state of the cilium to the trypanosome. We do hope that the reviewer agrees.

Part III – Minor Issues: Editorial and Data Presentation Modifications
Please use this section for editorial suggestions as well as relatively minor modifications of existing data that would enhance clarity.

Reviewer #1: Line 61: ‘underlie’
Thank you for catching this error. We fixed it in the revised text.

Line 144: ‘characterized by a higher’ (missing ‘a’)

Thank you for catching this error. The error was a missing ‘by’, not a missing ‘a’, but we fixed it in the revised text.

The introduction of FLAM3 knockdown is very brief and the unfamiliar reader might not appreciate the significant morphological change to which it gives rise. It would be good to expand the text a little around line 209 given the central utility of this modification.
We thank the reviewer for this suggestion. We agree (as does Reviewer 2) that the original version of the manuscript lacked in the rationale behind the FLAM3 knockdown. So, in response to this point and a couple of the points from Reviewer 2, we edited this section (around line 209 of the original manuscript) to clarify the whats and whys around the FLAM3 knockdown. We also included clarifying text in the introduction and multiple other locations throughout the manuscript to clarify the effects of FLAM3 KD. We hope that the reviewer agrees that the revised manuscript is much clearer in regard to FLAM3.

Could the authors be a bit clearer about the nomenclature in line 245? How about something like: “‘NN’ and ‘MK’ are used to represent cells with N nuclei and M kinetoplasts”? Otherwise the reference to N>2 is a bit confusing considering that you talk about 2K1N later in the figure caption.
We agree with the reviewer that this is a bit confusing. We clarified the nomenclature by defining cells as having x kinetoplasts (xK) and y nuclei (yN), and when cells have multiple (>2), we use M. For example, a cell with 4 kinetoplasts and 4 nuclei would be classified as MK MH because it has >2 kinetoplasts and nuclei. We hope that this clarifies the situation. We made the changes in the caption to Figure 3 to address this issue.

In the optical tweezers assay, Fig. 6 is a bit confusing. The cartoon in Fig. 6A and Fig. 6B show a cell with its anterior-posterior line more-or-less in the focal plane. Movie S1A shows the cell oriented at 90 degrees to this and aligned with the laser beam axis (you can see the anterior tip far from the focal plane, I’d guess towards the observer). The latter is what I would expect for a cell trapped for any length of time; it has a higher refractive index than its environment, so the cell body ‘wants’ to sit inside the laser beam as much as possible. This would tally with the 1-2Hz frequency observed, and it seems. Is that right, and if so, could you correct the figure?
We agree with the reviewer that the cartoon in Fig. 6A is a bit confusing. We agree with the reviewer’s interpretation of the movies and the physical interpretation of what happens to a cell in an optical trap. The trapped (posterior) part of the cell would be below the trap center (towards the observer in our inverted microscope) due to scattering force. This is also shown in S1B Movie, where the cell is brought to and lays flat on the surface. We edited Fig 6A by orienting the cell with cell body aligned to the laser and the anterior end of cell up.

The image in Fig 6B was taken by bringing the trapped cell near to surface. We did the for two reasons. First, it shows the trap position (as the laser reflects off the cover glass water interface) and constrains the cell to lie flat on the surface with focus plane. These two aspects of the image enable us to locate the position of the trap on the trypanosome’s cell body. We edited the caption of Fig 6 to clarify this point.

The figure numbering isn’t consistently referenced. In line 484 (and subsequently), the authors refer to Fig.7 when I think they mean Fig. 5. Could they check that the figures are referenced correctly?

We thank the reviewer for finding this error. The figure numbering was incorrect in multiple locations, but we have now corrected it everywhere.

The use of ‘velocity’ and ‘speed’ are misleading, as these are (mathematically speaking) vector and scalar versions of the same quantity. The authors could leave ‘speed’ as is, but replace ‘velocity’ with ‘total displacement after 10 seconds’, modifying the relevant quantities
We agree with the reviewer that our use of the terms ‘velocity’ and ‘speed’ were unclear in the original manuscript. We more clearly defined the terms in the materials and methods section and applied that definition faithfully throughout the manuscript. Additionally, we ensured that we pointed the reader to the definition in the materials and methods section at first mention and throughout the manuscript, where appropriate.

We believe that the confusion about these terms stems from our looseness with the language in the original version of the manuscript by using ‘speed’ and ‘velocity’ rather than everywhere using ‘average speed’ and ‘average velocity’. The magnitude of the average velocity (magnitude of the total displacement divided by total time) is a different value than the average speed (path length divided by total time), while the instantaneous speed (found by taking the limit of average speed as the total time goes to zero) is equal to the magnitude of the instantaneous velocity (found by taking the limit of the magnitude of the average velocity as the total time goes to zero). We have only calculated average velocity and average speed in the manuscript, and we have clarified this throughout the manuscript. We hope that our mitigation of this confusion is acceptable to the reviewer, as an alternative to the reviewer’s explicit suggestion, because it is a more general quantification of motility than ‘total displacement after 10 seconds,’ which is dependent on the somewhat arbitrary choice to use 10 seconds of tracked data.

Please soften the statement in line 508-510; the ciliary waveform is presumably different in LC2 knockdown, and could be argued to give a smaller net force (as is observed through lower net speed).

We agree with the reviewer that waveform differences we show in Fig 8 could influence the force. Therefore, we softened the statements in lines 508 – 510.

Line 609: I’d suggest 'bias' (rather than ‘biasedness’)

We edited the text, as suggested by the reviewer.

The data in lines 616-619 would be better presented in a table, rather than in-line.

We thank the reviewer for this suggestion. We compiled all the parameters in a table (Table 1). This is a clearer way of presenting the results.

Lines 731-733: Chlamydomonas cells rely on balancing the torque from one flagellum with that from the second in order to move in a straight line; removing one flagellum doesn’t change the direction of the beating wave, it makes cells rotate more-or-less in place because the torque isn’t balanced any more. I’d suggest revising this sentence. Also, it’s interesting to note that Chalmydomonas *do* occasionally have a quasi-symmetric beating mode – the so-called ‘shock response’ (e.g. Rüffer and Nultsch, Botanica Acta 108 p.255, 1995).

We agree with the reviewer that this section of the text was unclear. We clarified the text as suggested by the reviewer, and we included the reference to the light shock response and Rüffer and Nultsch, 1995.

Line 770: I liked the experiments where the samples were agitated, and this adds weight to the hypothesis about cell replication, but the *local* fluid shear rates in an agitated culture are probably much less than those experienced by a cell as it tries to break free from a neighbor so I’m not surprised that the agitation wasn’t sufficient to release cells.
We thank the reviewer for pointing out this potential issue with our assumptions. We performed an order of magnitude estimate on the shear stress due to swirling in the culture flask, where $\tau_{\text{flask}}=a\sqrt{\eta\rho\left( 2\pi f \right)^{3}}$ [Warboys et al. *Atherosclerosis* 285, 170–177 (2019)]. Assuming

- a = orbital radius of rotation of the shaker (0.95 cm)
- ρ = density of the culture medium (0.9933 g/mL)
- η = viscosity of the medium (0.0101 poise = 0.001 pN/μm^2^)
- *f* = frequency of rotation (90 rpm or 1.5 rps)

We found that $\tau_{\text{flask}}\sim0.3 \frac{\text{pN}}{\text{μm}^{\text{2}}}$

Additionally, we performed an order of magnitude estimate on the ciliary beat-induced shear stress on a swimming trypanosome, $\tau_{\text{swim}}\sim\frac{3}{2}\frac{\eta U}{R}$. Assuming

- η = viscosity of the medium (0.0101 poise = 0.001 pN/μm^2^)
- U = swimming speed of a trypanosome cell (20 μm/s)
- R = characteristic radius of a trypanosome cell (1 μm)

We found that $\tau_{\text{swim}}\sim0.03 \frac{\text{pN}}{\text{μm}^{\text{2}}}$.

So, the shear stress exerted by the orbital shaker is more than enough to mimic the effect of beating cilia on final stage of cytokinesis.

We added clarifying statements in the discussion section and added this order of magnitude analysis to the supporting information.

Lines 842-845: I agree that detaching the cilium from the cell body makes it easier to see what’s going on, but the ciliary waveform is the product of dynein activity and passive loads, including the PFR and the viscous load of the cell body. In this light, it seems unlikely that a FLAM3 knockdown simply ‘amplifies’ the flagellar waveforms – the flagellum is under different mechanical constraints, and it’s not clear how general these results will be.

We agree that the biophysical consequences of the FAZ and thus FLAM3 knockdown are complex and unlikely to simply “amplify” the flagellar waveforms by increasing the physical amplitude of the waveform. Instead, what we are saying is that the effect of FLAM3 knockdown makes the cilium more susceptible to various physical and genetic perturbations. We addressed this issue in some detail in the “The effect of TbLC2 knockdown on cell morphology and motility is more pronounced in FLAM3 KD than in wild-type parental cells” section of the Discussion.

Reviewer #2: Line 196-197: To characterize the localization of LC2, one could use antibodies against axonemal components, such as pan-centrin antibody 20H5, for a direct comparison.
We thank the reviewer for this suggestion. We added a panel to Fig 2 showing the anti-ɑ-tubulin (DM1A clone antibody) and AF 594 secondary antibody in FLAM3-LC2 KD/LC2 OE cells to stain the axoneme, along with the entire cell body. The confocal imaging shows colocalization of the eGFP signal from LC2::eGFP and AF 594 signal of ɑ-tubulin along the cilium. We updated the text in the results section, as well as added details in the methods, to support this change.

Line 213: Considering that pXS2 employs the PARP promoter, which is the strongest native promoter known in proyclics, it is very likely that LC2-eGFP levels dwarf the endogenous pool of untagged protein- this could be checked by qPCR at least. It is hard to explain why the eGFP-tagged protein doesn't incorporate well in to the axoneme in the presence of the WT protein. Do the authors show that this is actually the case?
We thank the reviewer for bringing this to our attention. However, as we discussed above, we never use the pXS2 plasmid for anything other than a convenient intermediate vector from which to PCR amplify the assembled TbLC2.BCCP.His6/eGFP chimeric DNA in this work. We never use it to express LC2 in trypanosome cells in any of the data presented here. We clarified the extent to which we used the pXS2 plasmids in the materials and methods.

In any event, it is not so much that the eGFP-tagged protein doesn't incorporate well in to the axoneme in the presence of the WT protein, as it is difficult to distinguish (see Fig 4A). We wanted to maximize our ability to resolve the localization of LC2-eGFP, therefore we knocked down the endogenous TbLC2. We edited the text around line 213 in the original manuscript to clarify this point.

Line 209-219: Could more directly check for coloc of LC2 with axoneme with detergent extraction of cells, to remove cytosolic pool, then stain cells for an axonemal marker protein.
As we discussed above, we added a panel to Fig 2 showing the anti-ɑ-tubulin (as an axoneme marker because it is so rich in tubulin) stained FLAM3-LC2 KD/LC2 OE cells to stain localize LC2 to the axoneme and not the PFR. The FLAM3 KD effectively spatially separated the cilium from the cell body, so there was no need to remove the cytosolic pool. We updated the text in the results section, as well as added details in the methods, to support this change.

Line 233: What is the rationale for studying the LC2 RNAi phenotype in the FLAM3 RNAi background? It makes sense in terms of their localization strategy, but the simplest thing would be to look at the effect of just LC2 RNAi before including the depletion of a second protein.
We again thank the reviewer for highlighting the importance of presenting the rationale behind using the FLAM3 RNAi background in this study earlier in the manuscript. We have included a paragraph at the end of the introduction to make the rationale clear – that to correlate mechanism of TbLC2 dynein regulation on the waveform with other cell biological and biophysical characterizes (including cell division phenotypes observed here in and around Line 233 and Fig 3 of the original manuscript) it is necessary to use the FLAM3 KD cells.

Line 273: The method used to describe "normal" DNA states does not account for dividing cells (1N2K, 2N2K), which frequently make up >20% of the population. Changes in the distribution of cell cycle stages should be reported and could reflect important aspects of the RNAi phenotype. Grouping everything that is not 1N1K into one category will miss a lot of important data.
As we discussed above, we edited the text and the caption of Fig 3 to clarify the presentation of the results and removed references to “severity” and “phenotype” of these classifications in an attempt to not correlate anything other than MK MN cells, which are associated with multi-ciliated amorphous cellular clumps (Fig 3D in revised manuscript), with abnormality. We clarified that other classifications like 2N 2K cells, for example, may be dividing, which is a perfectly normal process, in the caption to Fig 2, as suggested by the reviewer. We provided further analysis of the effects of TbLC2 knockdown on cell division by suggesting that cells that are neither 1K 1N nor MK MN may be dividing.

Line 293: The production of detached flagella in the LC2 phenotype suggests a defect in the formation of a new FAZ, which would cause issues with placement of the cleavage furrow. This would be an interesting result that arises from depleting a protein that localizes primarily to the flagellum. FAZ state could be checked with a FAZ antibody such as L3B2.
We agree with the reviewer that if we had seen detached cilia in LC2 knockdowns, this would have been an interesting result. However, we did not see any noticeable flagellar detachment in the LC2 knockdown cells (morphologically similar to the WT cells – we added a image of LC2 KD cell to Fig 2 to emphasize this point). Therefore, we have no indication that TbLC2 knockdown interferes with the production and integration of FAZ and thus we do not see any strong rationale to check the FAZ state with an antibody such as L3B2 on LC2 KD cells, for example. We hope that the reviewer agrees.

However, we did use FLAM3 knockdown cells as a background in our study of the effect of LC2 knockdown in microscopic motility, as we discussed in above. This has been shown in multiple of the references we provided in the manuscript to disrupt the FAZ. Therefore, we suggest that further analysis of the effect of FLAM3 KD on the FAZ in this manuscript is unnecessary. We hope that the reviewer agrees.

Line 302: Why is this analysis being done in the FLAM3/LC2 dual RNAi, if you are just trying to look at the function of LC2?
We again thank the reviewer for highlighting the importance of presenting the rationale behind using the FLAM3 RNAi background in this study earlier in the manuscript. As we discussed above, we have included a paragraph at the end of the introduction to make the rationale clear – that to correlate mechanism of TbLC2 dynein regulation on the waveform with other cell biological and biophysical characteristics it is necessary to use the FLAM3 KD cells.

Previous studies, including those we have now cited in the revised version of the manuscript, have shown that the detachment of the cilium caused by FLAM3 knockdown has no effect on the preassembly of the ciliary dynein complexes in the cytoplasm or their incorporation into the axoneme. Moreover, FLAM3 knockdown has not been shown to affect the entry or transport of axonemal components into and from the cilium. The fully functioning transport system was also validated by our localization of LC2::eGFP into the axoneme. So, in terms of the overall axoneme architecture, the wild-type and FLAM3 knockdown cells likely have identical characteristics. Therefore, because our ciliary tracking and waveform analysis are significantly aided by the use of FLAM3 knockdown cells with detached cilia, we opted to use FLAM3-LC2 double knockdown cells, instead of just LC2 knockdown cells, in the TEMs. We added a couple statements summarizing this rationale to the revised manuscript around Line 302 of the original manuscript.

Line 384-385: It should be noted that a cell with 3 kinetoplasts and one nucleus would be aberrant under any circumstance, not just in terms of their arrangement within a single cell.
We agree with the reviewer and, as we discussed above, we clarified the statements we made around the mislocalization and number of kinetoplasts and nuclei in multiple places in the manuscript, including in and around line 385-384 of the original manuscript.

Line 350: The similarities in the growth rates at early stages could be attributable to delays in the turnover of the LC protein. This could be tested by western blotting of the RNAi and control samples to look for when the LC2 levels decline. If tagging is an issue qPCR could be used as an alternative to look for loss of mRNA.
We agree with the reviewer that the similarities in the growth rates at early stages could be due to delays in target protein turnover after induction. The lag in the growth phenotype exhibited by RNAi induced cells is commonly seen in trypanosome protein knockdowns, including the one observed in the cilium with LC1 KD [Baron et al 2007], RNA Pol [Wang et al 2000], and mitochondrial Letm1 [Hashimi et al, 2013], for example. We did not quantify this effect for multiple reasons, including the lack of antibodies against the target proteins, the strong evidence for this conclusion in the literature, but perhaps most importantly, the cause for the delay in observing the phenotype would not change the principal conclusions and so the test would lengthen an already long (sorry about that) manuscript. We clarified this statement and provided these references in the manuscript.

Line 354: I find it curious that there is no change in growth rate between the shaken and unshaken FLAM3/LC2 RNAi cells, considering that the shaking limited the amount of cell clustering. How were the cell counts being done?
We thank the reviewer for pointing out this curiosity. We observed only a minimal reduction in cluster formation in the FLAM3-LC2 KD cell cultures by shaking during incubation (Fig 3). Because the reduction in cluster formation was not so strong, we suggest that the data in S6 Fig supports to the idea TbLC2 knockdown has an indirect, ciliary beating waveform-related, effect on cytokinesis, as opposed to simply a swimming shear stress-related effect (as we discussed in our response to a similar point made by Reviewer 1). We monitored the cell growth rate using a cell counting chamber to find the cell density every 24 hours. We clarified the manuscript both in the discussion section around S6 Fig and provided more detail the methods section around this point as well as a section about the relative magnitudes of shear stress in the supplement.

Line 379-380: In terms of LC2, what is the difference in expression levels between the "LC2 OE" and "Rescued" cells? Are they both pXS2 overexpressors? The nomenclature here is not clear.
We thank the reviewer for pointing out the lack of clarity around overexpression vectors. All overexpression cell lines used in this study are pLEW100V5 overexpressions. We clarified this point in multiple locations within the results (including around lines 379-380), the discussion and materials and methods sections, as well as the supporting information.

Line 378: Is this overexpression constitutive or tet-inducible? It's not clear but since they are talking about induction in later paragraphs I assume it's tet-inducible? From checking the M&M I realize that there are pXS2 and pLEW100 versions of the overexpression being used- it should be made very clear to distinguish between the two. If a pXS2 O/E is being compared to a pLEW100 in this case, an anti-GFP western could be used to see if one is expressing more than the other. In my experience, pLEW100 O/E tend to be higher than a constitutive O/E like pXS2 because with tet induction long-term cell viability is not necessary, so you can achieve levels of protein expression that you can't with constitutive expression.
We thank the reviewer for highlighting the confusion around and alerting us to some of the differences between the pXS2 vs pLEW100 expression plasmids in our manuscript. As we discussed above, we only used the tet-inducible pLEW100V5 overexpression vector in this study. We clarified this in multiple locations in the text, and hope that the reviewer agrees that the revised version of the manuscript is clearer now.

Line 1003-1010: From digging in the materials and methods I now see that the depletion of LC2 was checked using qPCR. This should be mentioned in the results section. Parts of the M&M read more like a Results section- I don't think there should be data here that is not mentioned in the rest of the work?
We thank the reviewer for pointing this out. We moved first mention of these data to the results section.

Line 448: "Overexpressing TbLC2::eGFP in the FLAM3/rescued cells..." Is this pXS2 overexpression that is implied by "rescued" or some additional overexpression? This is very confusing.
We thank the reviewer for highlighting the confusion around the pXS2 vs pLEW100V5 expression plasmids in our manuscript. As we discussed above, we only used the tet-inducible pLEW100V5 overexpression vector in this study. We clarified this in multiple locations in the text, and hope that the reviewer agrees that the revised version of the manuscript is clearer now.

Line 504-507: If the overexpression of the LC2::eGFP is causing motility defects, I don't understand how it can be called a "rescue" control. Technically, an equally reasonable interpretation is that the RNAi has an off-target effect that cannot be compensated for by the eGFP allele.

We thank the reviewer for highlighting the confusing use of the term “rescue” in our manuscript. It is true that the overexpression of LC2::eGFP causes motility defects, so this is, indeed, not a true rescue control. However, we have seen a significant, if not full, restoration of multiple phenotypes, including growth and motility, in the in LC2::eGFP expressing, FLAM3-LC2 KDs cell. In the revised manuscript we removed all instances of calling overexpression of the LC2::eGFP a “rescue” in description as well as nomenclature, in any cell line. We hope that the reviewer agrees that the revised version of the manuscript is clearer.
