# Supplementary material for: Light chain 2 is a Tctex-type related axonemal dynein light chain that regulates directional ciliary motility in Trypanosoma brucei
Source: PLoS Pathog. 2022 Sep 26;18(9):e1009984. doi: 10.1371/journal.ppat.1009984 (PMC9536576; doi:10.1371/journal.ppat.1009984)
Supplement: Attachment — Submitted filename: Godar et al. Response to reviewers June2022.docx [file ppat.1009984.s029.docx]

**Part I - Summary**

We thank the reviewers for another careful reading of the manuscript and constructive suggestions that we believe have strengthened the manuscript greatly. Please see our replies to each issue in blue below and highlighted in yellow in the manuscript.

Reviewer #1: This paper describes the structure and function of a light-chain dynein-related protein in Trypanosoma brucei that the authors refer to as TbLC2. The authors investigate its localisation and model its structure with a variety of standard methods. The effect of TbLC2 on motility is described at some length, in a series of experiments in which the authors use FLAM3 knockdown cells to enable better visualisation of flagellar waveforms. The role of TbLC2 in regulating flagellar beating is discussed and compared to the role played by structurally similar proteins in other model microorganisms such as C. reinhardtii. (comments on novelty and significance are unchanged from my previous review - the work is novel, significant and well-executed)

We thank the reviewer for the careful reading of the manuscript.

Reviewer #2: See below

**Part II – Major Issues: Key Experiments Required for Acceptance**

Reviewer #1: None

Reviewer #2: In this revision, the authors have addressed some of the points brought up during the original review, but I have substantial concerns about the work as it is currently formulated. First and foremost, the clarification about the use of the FLAM3 co-RNAi to study the LC2 RNAi phenotype makes sense for the laser tweezer and perhaps some of the motility assays- it is necessary to unmask the flagellum from the cell body. However, no justification is provided for the study of the LC2 FLAM3 co-RNAi for the cell division and morphology data provided in the paper.

We thank the reviewer for pointing out the lack of clarity in our justification for the use of FLAM3 and LC2 co-RNAI knockdown cell line in some of the analyses performed in the manuscript, especially in the results related to cell division and morphology. We modified the manuscript, as suggested by the reviewer’s subsequent comments, by removing the details on the cell division and morphology from the main text, as detailed below.

FLAM3 is a component of the flagellum side of the FAZ; LC2 is an outer arm dynein- these two structures are not likely to be in contact with one another or have a direct effect on each other- the authors do not provide any evidence for this. The idea appears to revolve around synthetic effects that arise when both proteins are depleted.

The idea that the FLAM3 protein and the LC2 protein are not likely to be in contact with one another or have a direct effect on each other is strongly supported by the structural evidence we discussed in detail and clearly cited in the main text of the manuscript.

The main reason for including the FLAM3 RNAi appears to be that it exacerbates the very mild effects of LC2 RNAi- considering that there is no obvious or proposed connection between these proteins, it is difficult to understand how this is justified- it is certainly not explored in this paper.

As we discussed in detail, clarified in multiple locations, and provided citations for from the literature in the main text, the reason we used the FLAM3 knockdown was two-fold.

First, FLAM3 enabled us to perform a detailed, quantitative study of the effects of LC2 knockdown on the beating waveform of the cilium, which is not technically feasible (due to imaging and image analysis issues, as discussed in the manuscript) without it. To directly correlate the effects observed in these waveform assays to all the other assays we report in the manuscript, we used the FLAM3 KD background in conjunction with WT cells, when possible.

Second, the anterior part of the cilium wraps around the cell body due to the FAZ, which provides a significant mechanical constraint to the cilium giving it a high effective stiffness. By knocking down FLAM3 and disrupting the mechanical constraints due to the FAZ, we lower the effective stiffness of the cilium. This lower effective stiffness increases the physical response and deformation of the cilium due to the forces generated by dynein and the effects on this force regulation by LC2 making it easier to observe and possible quantify. Using multiple loading conditions (WT and a softer effective physical constraint) on the dynein motors allows one to reveal the somewhat subtle effects caused by biological perturbations that might affect motility, as we have done with like knockdown of dynein regulatory protein LC2.

We further clarified this justification in multiple locations throughout the main text of the revised manuscript in response to this critique by the reviewer. We hope the reviewer agrees that the justification is clearer, even if the reviewer may not have designed the experiments in the way that we have and reported here.

It is possible that performing RNAi on two other proteins with mild phenotypes would produce a stronger effect, which may arise due to complex pleiotropic effects. The authors attribute the stronger effects of the dual depletion as strictly unmasking hidden function of LC2, showing its true importance, when the same case can be made for FLAM3. If they are interested in the synthetic effect, they need to consider the contributions of both proteins.

There is always the chance when one knocks down one or more proteins that there are pleiotropic effects on the cell biological and cell biophysical phenotypes. This is always the case in cell biology. However, we do not agree with the reviewer that the case could be made that knocking down LC2 could unmask the true importance of FLAM3. To do so would ignore strong evidence in the literature about the role of FLAM3, the biophysical constraints imparted by the cell body on the cilium due to the FAZ, multiple other careful quantifications of the effect of FLAM3 KD on the cell biology and cell biophysics of trypanosome cells, and the fundamental physics and mechanics that underly the motility of trypanosome cells. As we mentioned above, we have clarified the justification for using FLAM3, considering the contributions of both proteins, in each of the assays throughout the manuscript, and we hope the reviewer agrees that the justification is clearer in the revised manuscript.

I strongly suggest that the authors remove all of the cell division and morphology data from the paper that pertains to the dual FLAM3/LC2 RNAi. This would significantly streamline the paper- I will try to propose what could be shortened or omitted below. The strongest part of the paper, and where the dual-RNA can be justified, is the analysis of the paper starting on line 449. The construction of the cell lines, localization of LC2, and the RNAi phenotypes specific to LC2 can all be included as a lead-in to this work.

We agree with the reviewer’s suggestion to remove all of the cell division and morphology data from the main text of the manuscript. Doing so has made the manuscript more clear and concise. Moreover, doing so did not weaken or alter any of the discussions or conclusions that we drew in this manuscript, while emphasizing the strongest parts of the paper. Therefore, we followed the reviewer’s guidelines in making these changes (detailed in part III of the review).

**Part III – Minor Issues: Editorial and Data Presentation Modifications**
Please use this section for editorial suggestions as well as relatively minor modifications of existing data that would enhance clarity.

Reviewer #1: The authors have significantly revised several aspects of their manuscript in this resubmission, and I thank them for clearly indicating the aspects that have changed. This is helpful in the context of a manuscript that is still rather lengthy: ~200 pages now, although I note that this includes the rebuttal. The clarification of the cell geometry in the optical tweezers experiment is great, and the additional context around Chlamydomonas motility is welcome. I still take issue with a couple of aspects, but these are now largely a matter of taste due to the authors' careful clarifications, and shouldn't prevent publication:

- The order-of magnitude shear stress calculation is interesting, but I'm not sure it's the right calculation. Warboys et al. (the reference cited) study the flow of fluid over a surface attached epthelial layer, but I believe that the T. brucei are in suspension? If so, that reduces the shear rate to tau= \eta du/dy, which for a 5 cm radius flask makes the shear stress ~0.01 Pa, or 1/3 of the stress experienced by a swimming cell. Let's leave that for the journal clubs to discuss though - the authors' argument is reasonable.

We thank the reviewer for the careful consideration of the order-of-magnitude analysis we have presented using the Warboys et al. approach. We believe in our interpretation of the fluid mechanics of this system. We would welcome a detailed discussion sometime with the reviewer, and we agree that it would be nice if journal clubs across the world dove into the details, too.

- I still think that the disctinction that the authors make between 'velocity' and 'speed' is unconventional, but the additional clarification describing how they define terms has helped. I was a bit confused by the statement "['average velocity'] is a more general quantification of motility than 'total displacement after 10 seconds,' which is dependent on the somewhat arbitrary choice to use 10 seconds of tracked data." The choice is a bit arbitrary, but that's what they're doing! Calling the quantity 'velocity' doesn't make it any less so, and presumably they would get different numbers if they used 5 or 20 seconds of data? At any rate, the definition is clear, so I'll leave it at that.

We thank the reviewer for the careful reading of the manuscript and for the consideration of the changes we made in the previous version. However, we disagree with the reviewer on the point that the distinction between velocity and speed is unconventional. The definitions and formulations we have used are the standard ones from any physics 101 textbook. Additionally, we would like to clarify that we are not calling the total displacement after 10 seconds velocity. Instead, we are dividing the magnitude of the total displacement by the 10 seconds that the cell took to undergo that displacement (see lines 1092-1093 in the revised manuscript). This normalization of displacement by the time yields velocity, which is a less arbitrary measure of motility than total displacement. Therefore, if we had calculated the average velocity after 5 seconds or 20 seconds, as the reviewer suggested, we would presumably get the same result as after 10 seconds (while the total displacement would certainly be different).

Reviewer #2: Line 143-161: The rationale for the FLAM3 RNAi belongs in the results, in context, not at the very beginning of the paper.

We agree with the reviewer that moving the rationale for the of use of FLAM3 RNAi cells to the results, in context, makes the manuscript clearer. Therefore, we removed the section from the introduction and blended it with the respective results.

Lines 153, 155: It would be better to say that FLAM3 RNAi produces T. brucei cells with epimastigote-like morphologies. rather than referring to them as "leishmania-like". Leishmania also have forms that lack extended flagella, so this terminology is vague.

We apologize to the reviewer for the confusion caused by referring the FLAM3 kd cells with partially detached cilia as “Leishmania-like”.

We agree with the reviewer and changed “leishmania-like” to” epimastigote-like” morphology in the manuscript as suggested.

Line 258-292: Omit the data that does not pertain directly to LC2 RNAi only. Therefore, there is no clustering phenotype. Unless you can specify why FLAM3 RNAi would exacerbate LC2 RNAi?

We agree with the reviewer that there is not a clear rationale for using FLAM3 RNAi cells in these assays at this point in the manuscript. We also agree that the manuscript benefits from the exclusion of this data from the main text. Therefore, we removed Fig. 3 from the main text and edited the sections that used the figure and the results. However, we believe that the phenotypes related to cytokinesis and cell morphology are of potential interest to some readers and do provide more detail on the rationale behind and consequences of using FLAM3 RNAi in the motility related experiments. So, we moved Fig 3 to the supplementary material (S7 Fig) and the subsection of text describing the figure to supplementary text (S1 Text).

Additionally, we also shifted the emphasis away from the cytokinesis and cell morphology defects and highlighted the effects of LC2 knockdown on dynein-based ciliary motility in the wild-type cells throughout the manuscript. We did provide a more detailed and clear description of why FLAM3 RNAi would exacerbate LC2 RNAi with references to the literature in multiple places in the manuscript, as described above.

Line 293: "basal body duplication followed by nuclear and kinetoplast DNA replication initiates cilium replication"- this statement is unclear.

We thank the reviewer for pointing out the issue with the wrong choice of the word in the text. We changed the word initiates to precedes and believe that this change clarifies the statement.

Lines 300-308: LC2 RNAi on its own does not cause the appearance of aberrant DNA states. It is unclear what the authors are specifically talking about in Fig 3B- one image is just a DAPI channel- is this the RNAi case, if so, which is the RNAi? The fact that one kinetoplast is too near one nucleus in what appears to be a 4N2K cell is picking a small defect out of a cell with much more severe issues. I would omit this section because LC1 RNAi on its own does not appear to cause DNA defects.

The reviewer is right in pointing out that knocking down LC2 only does not cause the aberrant DNA states. As per the reviewer’s suggestion and in conjunction with the discussion above, we removed the section related to the cytokinesis and cell morphology phenotypes around lines 300-308 from the main text. However, we did move it to supplementary text (S1 Text) for the potentially interested reader.

Moreover, the DAPI channel images in Fig 3B are not intended to represent specific cell lines. Instead, we included them to be representative of the various kinetoplast-nucleus conditions we used as a classification rule. To clarify it, we added a statement in the corresponding section of the supplement saying the cells are representative images of different N-K conditions observed in various cell lines used in the study

Line 301-311: The 1N1K cell state does not mean that the cells are not dividing- they almost certainly are. It just means that they have not segregated their DNA yet. Other earlier processes are likely to be ongoing.

We apologize to the reviewer and the readers for not making this clear enough in the earlier and current versions of the manuscript. We totally agree with the reviewer that they are dividing and the cytokinesis is not frozen in 1N1K state. In the revised supplement, we removed the text suggesting that the cells not dividing and replaced it with text indicating that the imaged cells are not actively in the state of mitosis at that particular instance.

Lines 320-338: LC2 RNAi does not cause any changes in cilia length. FLAM3 KD and FLAM3-LC2 KD RNAi cilia are slightly shorter, but there was no difference between them. Since there's no LC2-related phenotype, omit. TbCL2 RNAi does not disrupt cell division. Can be omitted.

We agree with the reviewer that LC2 knockdown does not seem to disrupt the ciliary neogenesis. However, this result, combined with the SEM results, rules out the possibility of any structural defect on the flagellum incurred by the knockdown. Also, the fact that the flagellar lengths are same between the FLAM3 KD cells and the FLAM3-LC2 KD indicates that the motility phenotypes observed, specifically the parameters of the beating waveform, are not associated with variation in ciliary lengths. In sum, because there is no LC2 phenotype, these results strengthen our hypothesis that LC2 regulates the dynein-based motility in the cell. Additionally, as mentioned above, we removed the entire result around Fig 3 to supplementary material and text. We hope the reviewer agrees with us that despite the “negative result” on the ciliary length, the result is an imortant one to report and that the reviewer sees the benefit of keeping this portion in the revised manuscript.

Line 381: The difference in growth rates between control and LC2 RNAi cells is very small. I would be very cautious in claiming a difference in growth rate this small.

We agree that the reported difference in growth rates between the wild-type and LC2 RNAi cells does not reach the threshold of significance. Therefore, we removed the expression “slightly reduced rate” and modified the text around this line to reflect this change. We made similar changes around line 370 for similar reasons.

Line 393-412: Can be omitted.

We agree with the reviewer that this section of the manuscript has little to offer in terms of providing direct evidence of the implications of LC2 knockdown on wild-type cells. Therefore, We removed the portion (lines 393-402) as suggested by the reviewer. However, we kept lines 403-412 in the main text. We believe that lines 403-412 help us lay the foundation that the motility defect inflicted by the LC2 knockdown might have additional indirect and intricate effects on cytokinesis rather than a mere lack of shear force due to motility defect. We hope that the reviewer agrees with us in seeing the value of leaving this point in the manuscript.

Line 683-685: You cannot attribute these phenotypes to LC2 depletion alone- it's the synthetic effect of FLAM3 and LC2 depletion.

The reviewer is right that we cannot attribute the growth and cell division phenotypes to LC2 depletion alone in the wild-type cells. Therefore, we omitted the statement that describes the cytokinesis and morphology phenotypes being more prominent only in the FLAM3 RNAi background.

The reason behind keeping the growth and cell division phenotypes in the first place was to lay the foundation for investigating possible motility phenotypes in the LC2 knockdown cells. Many previous studies in multiple eukaryotes, including *C. reinhardtii*, have shown a direct correlation between disruption of flagellar dynein regulation to motility phenotypes, however, it is the unique flagellar and cell body architecture and physiology of the *T. brucei* cells that connects the flagella motility with the cell division and growth. Although we observed a significant growth, morphology, and motility phenotypes due to LC2 knockdown only in the background of FLAM3 knockdown cells, based on the characteristics of motility phenotypes that are typical to disruption in dynein regulation we propose that such phenotypes are due to LC2 depletion and its implication in dynein regulation and not just the synthetic effect of FLAM3 and LC2 depletion. As mentioned above, we have clarified our use of FLAM3 in multiple locations throughout the manuscript and have edited the manuscript as suggested, here.

Generally, the Discussion barely mentions the cell division and morphology data prior to line 449, which strongly calls for discarding many parts of it and focusing the motility data.

The reviewer is exactly correct – the discussion does barely mention the cell division and morphology data. Therefore, as we detailed above, we have removed much of it from the main text, but moved it to the supplement for the interested reader. We hope that the reviewer agrees that this has significantly improved the manuscript.
